# Transcriptomic Analysis and C-Terminal Epitope Tagging Reveal Differential Processing and Signaling of Endogenous TLR3 and TLR7

**DOI:** 10.3389/fimmu.2021.686060

**Published:** 2021-06-15

**Authors:** Chiung-Ya Chen, Yun-Fen Hung, Ching-Yen Tsai, Yi-Chun Shih, Ting-Fang Chou, Ming-Zong Lai, Ting-Fang Wang, Yi-Ping Hsueh

**Affiliations:** Institute of Molecular Biology, Academia Sinica, Taipei, Taiwan

**Keywords:** toll-like receptor, transcriptomic analysis, RNA-seq, epitope tagging, MYD88, signalosome

## Abstract

Toll-like receptor (TLR) signaling is critical for defense against pathogenic infection, as well as for modulating tissue development. Activation of different TLRs triggers common inflammatory responses such as cytokine induction. Here, we reveal differential impacts of TLR3 and TLR7 signaling on transcriptomic profiles in bone marrow-derived macrophages (BMDMs). Apart from self-regulation, TLR3, but not TLR7, induced expression of other TLRs, suggesting that TLR3 activation globally enhances innate immunity. Moreover, we observed diverse influences of TLR3 and TLR7 signaling on genes involved in methylation, caspase and autophagy pathways. We compared endogenous TLR3 and TLR7 by using CRISPR/Cas9 technology to knock in a dual Myc-HA tag at the 3’ ends of mouse *Tlr3* and *Tlr7*. Using anti-HA antibodies to detect endogenous tagged TLR3 and TLR7, we found that both TLRs display differential tissue expression and posttranslational modifications. C-terminal tagging did not impair TLR3 activity. However, it disrupted the interaction between TLR7 and myeloid differentiation primary response 88 (MYD88), the Tir domain-containing adaptor of TLR7, which blocked its downstream signaling necessary to trigger cytokine and chemokine expression. Our study demonstrates different properties for TLR3 and TLR7, and also provides useful mouse models for further investigation of these two RNA-sensing TLRs.

## Introduction

Innate immunity is the first line of defense for eliminating pathogens. To detect diverse pathogens, hosts have evolved various pattern recognition receptors (PRRs) to recognize foreign molecules derived from pathogens (1, 2). Toll-like receptors (TLRs), some of the best studied PRRs, are critical for detecting bacterial and viral molecules and for triggering expression of downstream target genes, including cytokines and chemokines, to activate an inflammatory response and remove pathogens (3–5).

Of the 13 recognized TLRs, TLR3, TLR7, TLR8 and TLR9 are known as nucleic acid-sensing TLRs (4, 6, 7). TLR3 responds to double-stranded RNA (dsRNA), whereas TLR7 and TLR8 both recognize single-stranded RNA (ssRNA) (8–10). TLR9 binds to demethylated DNA, mainly derived from bacteria and viruses (11). In addition to detecting the DNA or RNA of pathogens, nucleic acid-sensing TLRs also recognize endogenous damage/danger signals such as self mRNA and DNA derived from dead cells from injured tissues (4, 12, 13), as well as micro RNAs (miRNAs) delivered by exosomes (14). Nucleic acid-sensing TLRs are subjected to multiple regulatory mechanisms, such as intracellular localization, proteolytic processing, as well as ligand processing and recognition, which together control their activation by the partially digested nucleic acids from pathogens and other endogenous ligands (6, 7, 15).

After translation of nucleic acid-sensing *Tlr* mRNAs, the trafficking chaperone Unc93b1 controls their transport from the endoplasmic reticulum (ER) to endosomal compartments where they are proteolyzed into N- and C-terminal fragments (NTF and CTF) (6, 16, 17). The NTF and CTF of nucleic acid-sensing TLRs are still associated with each other for ligand recognition and signal transduction (18–20). Downstream signaling of TLRs is mediated by interactions between the cytoplasmic Tir domain of CTF and other Tir domain-containing adaptors (4, 5). The two best-studied Tir domain-containing adaptor proteins for TLRs are MYD88 and TIR domain-containing adapter inducing interferon-β (TRIF; also known as TICAM-1). Upon ligand binding, TLRs form a signalosome *via* interaction with MYD88 or TRIF to trigger cytokine expression (4, 5). In general, the association between TLRs and their downstream adaptor protein is mediated by interactions between the Tir domains, except for the interaction between TLR3 and MYD88 (4, 21). Instead of interacting with a Tir domain, TLR3 interacts with the death domain of MYD88 (21). Thus, when MYD88 binds TLR3, the Tir domain of MYD88 is still free to interact with other Tir domain-containing molecules. The MYD88-containing TLR3 signalosome is likely to be different from other TLRs. Consistent with this speculation, TLR3 acts *via* MYD88 to control neuronal morphology, but TLR3 also interacts with TRIF to induce cytokine expression in cultured neurons (21). Consequently, compared with other TLRs, TLR3 likely displays distinct protein-protein interactions with downstream signaling molecules, although more structure analysis is required to conclude this point.

Our previous study indicated that TLR3, TLR7 and TLR8 cell-autonomously control expression of distinct downstream target genes to regulate neuronal morphology (12, 21, 22). However, it is unclear whether these nucleic acid-sensing TLRs also have different target genes in non-neuronal cells, such as macrophages. Thus, we analyzed these nucleic acid-sensing TLRs by comparison their properties side-by-side in non-neuronal cells. Here, we first applied RNA-seq and transcriptomic profiling to confirm that downstream target genes of TLR3 and TLR7 are not identical, but partially overlapping in BMDMs. We then used CRISPR/Cas9 technology to dually tag Myc and HA cassettes at the C-terminal ends of endogenous TLR3 and TLR7 in mice. Anti-HA tag antibodies allowed us to analyze both TLR3 and TLR7 in immunoblotting, immunoprecipitation and immunostaining. Our comparison revealed the differences between TLR3 and TLR7 at multiple levels.

## Materials and Methods

### Animals

All mice were housed in the animal facility of the Institute of Molecular Biology, Academia Sinica, with a 12 h light/12 h dark cycle and controlled temperature and humidity. All animal experiments were performed with the approval of the Academia Sinica Institutional Animal Care and Utilization Committee and in strict accordance with its guidelines and those of the Council of Agriculture Guidebook for the Care and Use of Laboratory Animals (Protocol # 13-02-520). Both male and female mice were used randomly.

*Tlr3^–/–^* (10) and *Tlr7^–/y^* (23) mice in a C57BL/6 genetic background were purchased from Jackson Laboratory. To generate mice expressing C-terminal Myc-HA-tagged TLR3 (*Tlr3^t/t^* is used to represent homozygous tagged knockin mice), we adopted a CRISPR/Cas9-mediated knockin approach to insert the Myc-HA tag sequences into exon 7 of *Tlr3* before the stop codon. A gRNA (5’-tgcaagtagcacttggatct) and a single-stranded DNA template (ssODN: 5′-catgatggacctttataaattggatctatccctttaccgactccaaatcttcaaatgagttta**AGCGTAATCTGGAACATCGTATGGGTA**accgttCAGATCCTCTTCTGAGATGAGTTTTTGTTCTCTAGAatgtgctgaattTcTagatccaagtgctacttgcaatttatgatgaaaggcatttatccgttctttct) were used, incorporating an *XbaI* site (underlined capitals), as well as Myc (bold capitals) and HA (capitals) sequences.

To generate Myc-HA-tagged *Tlr7* mice (*Tlr7^t/y^* and *Tlr7^t/t^* are used hereafter to represent homozygous tagged knockin male and female mice, respectively), the Myc-HA tag sequences were inserted into exon 3 of *Tlr7* before the stop codon by using CRISPR/Cas9-mediated knockin approach. The gRNA (5’-ggcttatagtcaaatgttca) and ssODN (5’- cagaccttcatttatggaaacctttatgaaaacttcaggtaccaaggcatgtcctaggtggtgacattcttcagagagctaAGCGTAATCTGGAACATCGTATGGGTAaccgtt**CAGATCCTCTTCTGAGATGAGTTTTTGTTC** TCTAGAgactgtttccttgaacatttgactataagccacatgattgtctgtggtca) was used to incorporate an *XbaI* site (underlined capitals), as well as Myc (bold capitals) and HA (capitals) sequences.

The *Tlr7* gene in *Tlr7^t/y^* or *Tlr7^t/t^* mice also contains two loxP sites to flank the exon 3 that was achieved by using CRISPR/Cas9^D10A^ paired-nicking approach. Two pairs of gRNAs (intron2: 5’- accactgaagactttgataa and 5’- aagtacagtcacagggacgg; intron 3: 5’- ggccccaagaaaatgataag and 5’- gatgggttctcttggaaaat) and two ssODNs carried loxP sequences (capital) and HaeII site (underlined) (5’- tttgttagaaagctaagatggtacaagcaaaacataaaa**T**cattatcaaagtcttcagtggttaATAACTTCGTATAGCATACATTATACGAAGTTATggcgctataagtacagtcacagggacggaggtgctgtttacactattacaaacaagacctgtgttgtttagtttt; 5’- gcaatatgccacaaaagcagctactggtacaggacagttggtagctgcttcagtg**T**ctcttatcattttcttggggcccaggcgctATAACTTCGTATAGCATACATTATACGAAGTTATttgatgggttctcttggaaaatagggaagtttttttttgtcattcatgaacatggtcaacttaaaaatggggaaaatgga) were used along with Cas9^D10A^. The PAM sequences were mutated (bold capitals). The *Cas9* mRNA, *Cas9^D10A^* mRNA, gRNA preparation, and mouse production protocols were as described previously (24).

For genotyping, the *Tlr3*-tagged knockin and wild-type (WT) alleles were detected by PCR using Tlr3-Fw (5’-CACTCTGTTTGCGAAGAG-3’) and Tlr3-Rv (5’-CTATCCCTTTACCGACTC-3’) primers that amplify a 162 base pair (bp) fragment from the WT allele and a 232-bp fragment from the tagged knockin allele of *Tlr3*. To genotype *Tlr7^t/y^* and *Tlr7t/t* mice, we used Tlr7-Fw (5’-CATCAGAGGCTCCTGGATG-3’), Tlr7-Rv (5’-GCCCAGAGAACTTCTCAGTA-3’), and HA-Rv (5’-GCGTAATCTGGAACATCG-3’) to amplify a 708-bp fragment from the WT allele and a 293-bp fragment from the tagged knockin allele of *Tlr7*.

### RNA Sequencing (RNA-Seq)

BMDMs were treated with poly(I:C) or CL075 for 6 h. RNA-seq was performed as described previously (22, 25). Briefly, total RNA was extracted using Trizol, followed by DNase I digestion as described above. RNA quality and quantifications were determined using an Agilent 2100 Bioanalyzer. The mRNA sequencing libraries were prepared using a Truseq Stranded mRNA kit (Illumina), and 75–76 cycle single-read sequencing was performed using the 500 High-output v2 (75 cycle) sequencing kit on an Illumina NextSeq500 instrument. After removing adaptor sequences, the quality of raw sequences was examined in FastQC software. Bioinformatics analysis was performed using two systems. The first analysis was conducted using CLC Genomics Workbench (http://www.clcbio.com). Raw sequencing reads were trimmed of low-quality sequences (Phred quality score of < 20) and of sequences with length < 25 bp. Sequencing reads were mapped to the mouse genome assembly (GRCm38.p6) from Ensembl with the following parameters: mismatches cost=2, insertion cost=3, deletion cost=3, minimum fraction length=0.9, minimum fraction similarity=0.9, and maximum hits per read=5. A Generalized Linear Model (GLM) and Wald Test were then used to analyze differential expression of genes and their statistical significance. The second system employed Hisat2 for sequence mapping (GRCm38.p6) with default settings, featureCounts to count reads, and edgeR (qlf model) for differential expression and statistical analysis. To select significantly altered genes, we first set a false discovery rate (FDR) of P < 0.05 and fold change > 1.5. To remove genes with background expression levels, the average transcripts per kilobase million (TPM) in one of the groups must be > 0.5. Genes meeting the criteria for both analysis pipelines (CLC and Hisat2-featureCounts-edgeR) were considered as significantly differentially-expressed genes. In addition, noncoding RNAs were removed before gene ontology (GO) analysis using Metascape (http://metascape.org/gp/index.html#/main/step1). The raw datasets of the *Tlr3* and *Tlr7* target genes have been deposited online (NCBI GSE163347, https://www.ncbi.nlm.nih.gov/geo/query/acc.cgi?acc=GSE163347). Up- and down-regulated target genes of TLR3 and TLR7 are summarized in **Supplementary Table 1**.

### Primary Cultures of Neurons, Glial Cells and BMDMs

Neuronal culture containing both hippocampi and dorsal cortices was prepared at mouse embryonic day E16.5-17.5 as described previously (21, 22, 26). Mixed glia cultures were prepared from cerebral cortices and hippocampi of WT, *Tlr3^t/t^*, *Tlr7^t/y^*, and *Tlr7^t/t^* mice at postnatal day 2 (P2). The cells were cultured for 1 to 2 weeks in DMEM containing 10% fetal calf serum (FCS), penicillin and streptomycin. For BMDMs, bone marrow cells were collected from tibias and femurs of adult WT, *Tlr3^t/t^*, *Tlr7^t/y^*, and *Tlr7^t/t^* mice. The cells were then cultured in DMEM containing 10% fetal bovine serum (FBS), penicillin and streptomycin, and 50 μM β-mercaptoethanol plus 20% conditioned medium of L929 cells for 6 days. Poly(I:C) (10 μg/ml, InvivoGen), CL075 (4 μM, InvivoGen) and R848 (0.5 μg/ml, InvivoGen) were used to treat cells for TLR3 and TLR7 activation.

### Antibodies, Plasmids and Software

Detailed information on the antibodies used in this report is summarized in **Supplementary Table 2**. The mouse *Tlr3* cDNA encoding TLR3 protein (a.a. 1-905) was amplified by using the primer pair Tlr3-1Fw 5’ GGGATCCATGAAAGGGTGTTCC 3’ and Tlr3-2715Rv 5’ GCTGGTCGACATGTGCTGAATTCCG 3’, and then subcloned into pGw1-cMyc vector at the *BglII* and *SalI* sites to generate a TLR3-Myc expression construct. The mouse *Tlr7* cDNA encoding TLR7 protein (a.a. 1-1050) was amplified by using the primer pair Tlr7-1Fw 5’ GGGATCCGCCACCATGGTGTTTTCG 3’ and Tlr7-3150Rv 5’ GCTGGTCGACGACTGTTTCCTTGAAC 3’, and then subcloned into pGw1-cMyc vector at the *BglII* and *SalI* sites to generate a TLR7-Myc expression construct. All software used in this report is listed in **Supplementary Table 3**.

### Immunoprecipitation and Immunoblotting

For immunoprecipitation (IP), WT, *Tlr3^t/t^* and *Tlr7^t/y^* mouse brains and spleens were harvested and homogenized using a dounce homogenizer (20 passes) in ice-cold Tris-buffered saline (TBS; containing 20 mM Tris (pH7.4), 150 mM NaCl, 1 mM β-glycerophosphate, 1 mM PMSF, 1 μg/ml Aprotinin, 1 μg/ml Leupeptin and 1 μg/ml pepstatin). Proteins were further extracted by adding 0.5% Triton X-100 and gentle mixing at 4°C for 30 min. After removing cell debris by centrifugation, 250 μg lysate was incubated with 1 μg TLR3 (PaT3), TLR7 (A94B10), or HA antibody and 10 μl protein A/G slurry (1:1) at 4°C for 2-3 h. The resulting protein A/G resin was washed three times with TBS containing 0.5% Triton X-100 and then twice with 50 mM Tris (pH7.4). The IP complex was eluted by adding 1X SDS-sample buffer and boiling for 5 min. The samples were then subjected to immunoblotting analysis. To examine TLR7 signaling complex in BMDMs, WT and *Tlr7^t/y^* BMDMs were treated with 0.5 μg/ml R848 for 30 and 60 min at 37°C. After rinsing with cold PBS, the BMDMs were lysed with cold TBS containing 0.5% Triton X-100 and kept on ice for 10 min. After centrifugation, the BMDM extract was then used to carry out IP using TLR7 antibody (A94B10), as described above. Immunoblotting was performed as described previously (27). All full-size images of immunoblots are available in **Supplementary Figures 8** and **9**.

### Immunofluorescence Staining

Immunostaining of BMDMs and mixed glial cells was performed as described previously (27). Immunofluorescent images of cells were visualized at room temperature using a confocal microscope (LSM 700, Zeiss) equipped with a 63×/NA 1.4 objective lens (Plan-Apochromat) and Zen acquisition and analysis software (Zeiss). The images were processed using Photoshop (Adobe) with minimal adjustment of brightness or contrast applied to the entire images.

### RNA Extraction and Quantitative Real Time-PCR (RT-PCR)

To detect gene expression in BMDMs and glial cells, primary cell cultures were treated with 10 μg/ml poly(I:C) or 4 μM CL075 for 6 h before harvesting using TRIzol reagent. To quantify gene expression in spleen, adult WT and *Tlr3^t/t^* mice received an intraperitoneal injection of saline or 5 mg/kg poly(I:C). Six hours later, mouse spleens were harvested for total RNA extraction using TRIzol according to the manufacturer’s instructions (Invitrogen), followed by DNase I digestion (New England BioLabs). Reverse transcription and quantitative RT-PCR analysis was performed using the Transcriptor First Strand cDNA Synthesis Kit (Roche) with an oligo(dT)18 primer and the Universal ProbeLibrary probes (UPL; Roche) system. Primer sets and probe numbers for selected genes from the results of RNA-seq and internal control *Hprt* are listed in **Supplementary Table 4**. Relative expression levels of each gene were normalized to the levels of *Hprt* measured at the same time on the same reaction plate. Brain and spleen of adult WT and *Tlr7^t/y^* mice were also harvested and subjected to RNA extraction to enable quantitative RT-PCR analysis of *Tlr7* and *Tlr8* expression levels.

### Statistical Analysis

Statistical analyses were performed using GraphPad Prism 8.0 software. Experiments were performed blind by relabeling the samples with the assistance of other laboratory members. The majority of our data met the assumptions (normal distribution) for statistical tests. For two-group experiments, an unpaired t-test was used. For experiments with more than two groups, one-way ANOVA with *post hoc* Bonferroni correction was applied. For two-factor experiments, two-way ANOVA with *post hoc* Bonferroni correction was used. Data are presented as mean ± SEM. P values < 0.05 were considered significant.

## Results

### Different Downstream Target Genes of TLR3 and TLR7 in BMDMs

**Figure 1:**
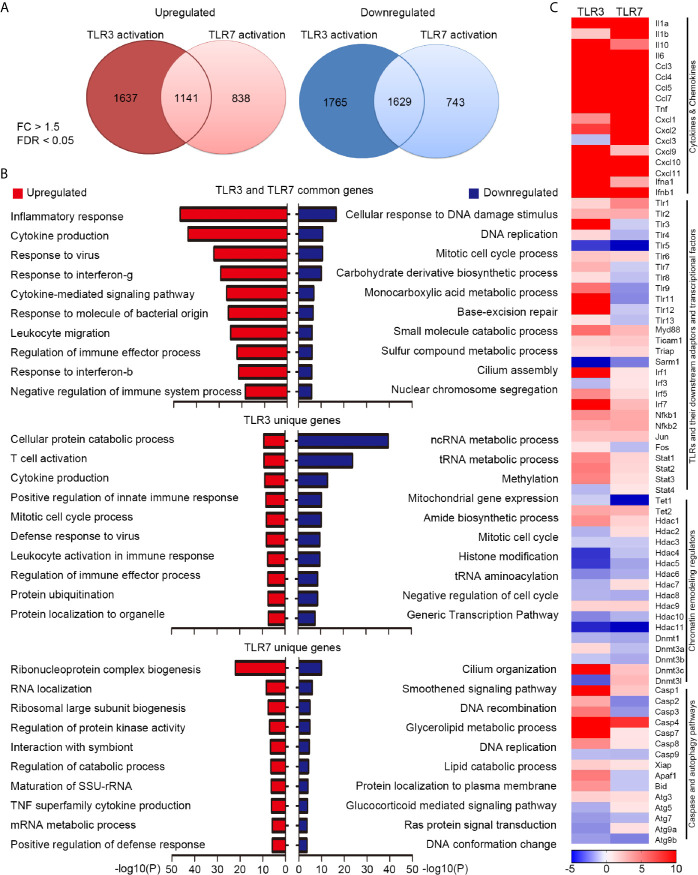
Transcriptomic profiling reveals differential downstream targets of TLR3 and TLR7 in BMDMs. **(A)** Venn diagram showing the overlap and difference between TLR3- and TLR7-regulated genes. Left: upregulated; Right: downregulated. Fold change (FC) > 1.5; false discovery rate (FDR) < 0.05. The numbers of identified genes are also indicated. **(B)** Gene ontology (GO) of commonly-targeted or uniquely-targeted TLR3 and TLR7 genes based on biological functions. Red bars: upregulated genes; blue bars: downregulated genes. The top ten GOs for each group are shown. **(C)** Heat map depicting the relative mRNA levels of selected TLR3- and TLR7-regulated genes. Scale bar: z-score.

Our previous study has indicated that TLR3 and TLR7 regulate expressions of distinct downstream genes to control cell morphology in neurons (21, 22). Since TLR3 and TLR7 both are involved in anti-virus response (28, 29), it is unclear whether TLR3 and TLR7 also trigger different transcriptomes in non-neuronal cells. To investigate this issue, we treated BMDMs with poly(I:C) or CL075 for 6 h to activate TLR3 and TLR7 respectively. The RNA samples were then subjected to RNA-seq. We set a fold-change (FC) > 1.5 and a false discovery rate (FDR) < 0.05 as criteria to analyze the target genes of TLR3 and TLR7. Under these conditions, we identified 2778 and 3394 genes upregulated or downregulated, respectively, by TLR3 activation in BMDMs (**Figure 1A**, **Supplementary Table 1**). Much fewer downstream target genes were identified for TLR7, with 1979 upregulated genes and 2372 downregulated genes (**Figure 1A** and **Supplementary Table 1**). Notably, 1141 upregulated genes and 1629 downregulated genes were commonly shared between the TLR3 and TLR7 datasets (**Figure 1A** and **Supplementary Table 1**). Gene ontology (GO) analysis using Metascape indicated that upregulated genes shared by the two proteins are highly relevant to innate immune responses, including inflammatory responses, cytokine production and signaling pathways, responses to viruses/bacteria, IFNγ and IFNβ, and regulation of immune processes (**Figure 1B**, upper panel, red bars; **Supplementary Table 1**). In terms of downregulated genes in common, the major GO terms related to DNA replication and damage responses and metabolic processes (**Figure 1B**, upper panel, blue bars and **Supplementary Table 1**). Gene lists of these GOs are summarized in **Supplementary Table 1.** These findings are consistent with the functions of TLR3 and TLR7 in triggering innate immunity and the physiological consequence of attenuated metabolic activity during inflammation (30).

Next, we performed Ingenuity Pathway Analysis (IPA) to further dissect the involvement of common target genes in canonical signaling pathways (**Supplementary Figure 1**), which identified more than 400 such pathways (–log(p) > 1 and ratio > 0.125). We summarize each top 50 pathways for up- and down-regulated target genes in **Supplementary Figure 1**. Details for five of those pathways are shown in **Supplementary Figures 2, 3**, including the role of pattern recognition receptors in recognition of bacteria and viruses, TLR signaling, activation of IRF by cytosolic pattern recognition receptors, IL-6 signaling, and the coronavirus pathogenesis pathway. These results further suggest that TLR3 and TLR7 activation control some common transcriptional factors (such as NF-kB, IRF3, IRF7, C-JUN, HIF1α, NF-IL6 and ISGF3) to induce expression of inflammatory and anti-viral cytokines (including IL-1β, IL-6, IL-10, IL-12, IL-17, TNFα/β, type I IFN) and chemokines (such as CCL2 and CCL5). Expression of other pattern recognition receptors and their downstream signaling adaptors and kinases could also be increased upon TLR3 and TLR7 activation (**Supplementary Figures 2, 3**), suggesting positive feedback regulation of TLR3 and TLR7 activation. These analyses further suggest that TLR3 and TLR7 share some common downstream pathways to trigger innate immunity.

Apart from the shared target genes, TLR3 activation actually uniquely impacted the expression of 3402 genes (1637 upregulated and 1765 downregulated) (**Figure 1A**). For TLR7, 838 genes were specifically upregulated and 743 were uniquely downregulated in BMDMs upon TLR7 activation (**Figure 1A**). GO analysis of these uniquely impacted genes indicated that, in addition to common pathways, TLR3 and TLR7 activation differentially regulates diverse cellular responses and processes (**Figure 1B**, middle and lower panels).

We further used a heat map to compare some of the common and uniquely impacted downstream genes of both TLR3 and TLR7, which included various cytokines and chemokines, other TLRs and their downstream adaptors and transcriptional factors, chromatin remodeling regulators, as well as caspase and autophagy pathways (**Figure 1C**). Among the various cytokine and chemokine genes, all except *Cxcl3* were upregulated by TLR3 or TLR7 activation, though to varying degrees (**Figure 1C**). In terms of the different *Tlr* genes, apart from upregulating *Tlr3* itself, TLR3 activation also increased expression levels of other *Tlr*s, apart from *Tlr5* (**Figure 1C**). In contrast, TLR7 activation only increased *Tlr1*, *Tlr2* and *Tlr6* expression, but it downregulated the expression of other *Tlr*s, including *Tlr3*, *Tlr4*, *Tlr5*, *Tlr8*, *Tlr9*, *Tlr11*, *Tlr12*, *Tlr13* and *Tlr7* itself (**Figure 1C**). Moreover, TLR3 and TLR7 activations had opposing effects on *Irf3*, *Fos*, *Stat4*, *Hdac2*, *Hdac7*, *Dnmt3a*, *Dnmt3l*, *Casp2*, *Casp3*, *Apaf1*, *Bid*, *Atg5* and *Atg9a* (**Figure 1C**). Thus, our transcriptomic profiling has clearly indicated that activations of TLR3 and TLR7 in BMDMs regulate the expression of shared and unique downstream genes. This outcome supports previous observations from neurons that endosomal TLRs, including TLR3 and TLR7, use differential downstream signaling pathways to control gene expression and cellular responses (22).

**Figure 2:**
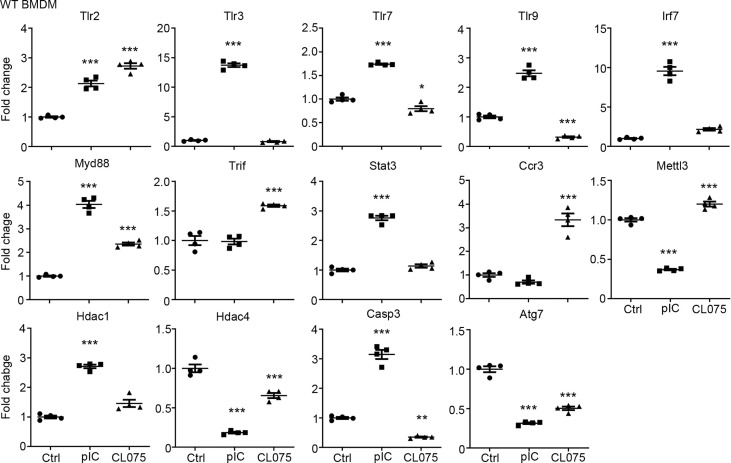
Quantitative RT-PCR confirms altered gene expression in response to TLR3 or TLR7 activation. Poly(I:C) and CL075 were used to activate TLR3 or TLR7, respectively, in WT BMDMs. The expression levels of indicated genes were determined by quantitative RT-PCR and normalized against internal control *Hprt*. Data are represented as mean ± SEM (*error bars*). Each dot indicates the data of one independent experiment. *P < 0.05; **P < 0.01; ***P < 0.001.

We then performed quantitative RT-PCR to confirm the results of our RNA-seq analysis. We selected 14 target genes from the lists of TLR3 and TLR7 target genes (**Figure 1C**) for further analysis. Overall, the results of quantitative RT-PCR were consistent with our RNA-seq data. For instance, the levels of *Tlr2* and *Myd88* were increased by both TLR3 and TLR7 activation. The levels of Hdac4 and Atg7 were reduced by both TLR3 and TLR7 activation. However, TLR3, but not TLR7, specifically increase the RNA levels of *Tlr3, Tlr7, Tlr9, Irf7, Stat3, Hdac1* and *Casp3* in BMDMs (**Figure 2**). Thus, both RNA-seq and quantitative RT-PCR indicate that TLR3 and TLR7 activations differentially induce expression of downstream target genes in BMDMs.

### Generation and Characterization of Myc-HA Knockin *Tlr3* and *Tlr7* Mice

We then tried to reanalyze the properties of TLR3 and TLR7 by comparing these two TLRs. We first deployed several commercially available TLR3 and TLR7 antibodies to detect the respective exogenous proteins in HEK293T cells, as well as the endogenous proteins in mouse spleen lysates, by immunoblotting analyses (**Supplementary Figure 4**). Among the three TLR3 antibodies we tested, only one antibody recognized overexpressed TLR3-Myc protein in HEK293T cells and none of them detected TLR3 in WT mouse spleen lysate (**Supplementary Figure 4A**). Only one of the tested TLR7 antibodies (eBioscience) recognized overexpressed TLR7-Myc, and it presented a stronger band at ~60 kDa in WT mouse spleen lysates compared with *Tlr7* knockout spleen (**Supplementary Figure 4B**). Unfortunately, this TLR7 antibody from eBioscience also detected several nonspecific bands and has been discontinued. It is difficult to examine expression profiles and the function of endogenous TLR3 and TLR7 proteins when validated antibodies are not available.

**Figure 3:**
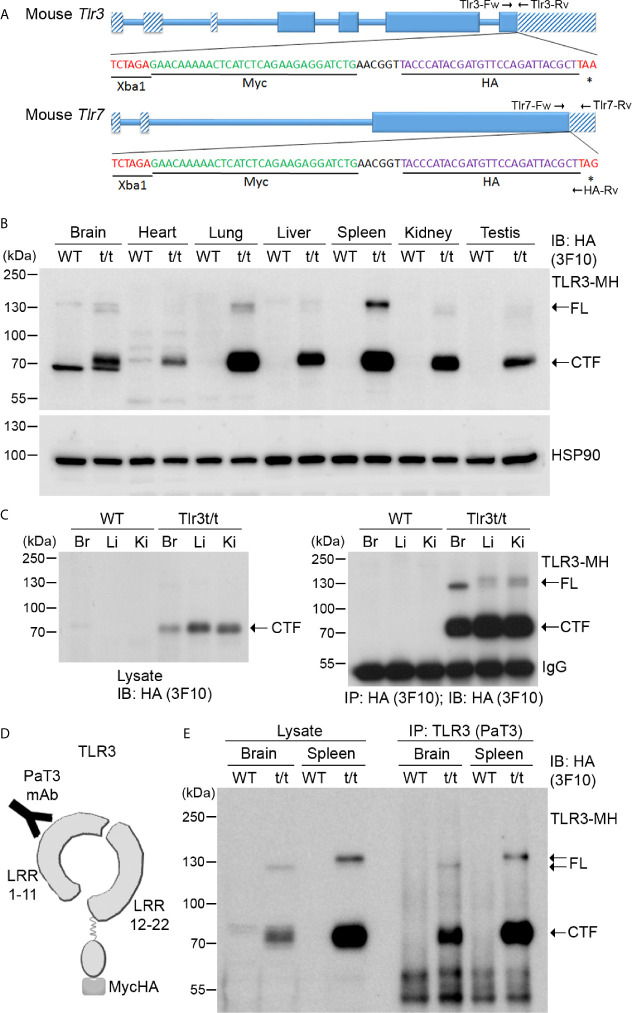
Dual tagging of Myc and HA cassettes at the C-terminal ends of the *Tlr* genes reveals expression and processing of TLR. **(A)** Schematic of the mouse *Tlr3* and *Tlr7* genes in which a Myc-HA epitope tag has been inserted before the stop codon. Two primer sets, i.e. Tlr3-Fw and Tlr3-Rv for *Tlr3* and Tlr7-Fw, Tlr7-Rv and HA-Rv for *Tlr7*, were used for genotyping the tagged *Tlr3* and *Tlr7* mice. The results of genotyping are available in **Supplementary Figure 2**. *, stop codon. **(B)** Detection of TLR3-MH protein in multiple tissues of *Tlr3^t/t^* mice using immunoblotting (IB) with anti-HA antibody (3F10). HSP90 was used as a loading control. **(C)** Anti-HA antibody (3F10) was used in immunoprecipitation (IP) of TLR3-MH from brain (Br), liver (Li) and kidney (Ki) of WT and *Tlr3^t/t^* mice. The IP complex was then analyzed by IB with the same anti-HA antibody. **(D)** Schematic of TLR3 protein with the C-terminal dual Myc-HA tag. The PaT3 monoclonal antibody recognizes the N-terminal region of TLR3. **(E)** The N-terminal (NTF) and C-terminal (CTF) fragments of TLR3 remain associated with each other after proteolytic cleavage. TLR3 was precipitated using the PaT3 antibody from brain and spleen lysates of WT and *Tlr3^t/t^* mice. The IP complex was then subjected to IB analysis using anti-HA antibody (3F10). FL, full-length TLR3-MH; CTF, C-terminal fragment of TLR3-MH; IgG, immunoglobulin heavy chain.

To overcome the antibody issue and to allow us to investigate expression and functions of the TLR3 and TLR7 proteins *in vivo*, we added epitope tags at the 3’ end of the *Tlr3* and *Tlr7* genes to monitor expression of endogenous TLR3 and TLR7 proteins using commercial anti-tag antibodies. We applied a CRISPR/Cas9-mediated knockin approach to insert a sequence containing both Myc and HA tags before the stop codons of the *Tlr3* gene in C57BL/6 mice (**Figure 3A** and **Supplementary Figure 5A**). Insertion of the dual Myc-HA tag was confirmed by genomic PCR and sequencing (**Supplementary Figure 5A**). Dual Myc-HA tagging did not seem to influence mouse growth or development as the appearance of knockin mice was comparable to their wild type (WT) littermates (**Supplementary Figure 5B**). We applied the same approach to generate and characterize knockin mice with a Myc-HA tag at the TLR7 C-terminal tail (**Figure 3A** and **Supplementary Figures 5C, D**). Hereafter, for simplicity, we name these knockin models as *Tlr3^t/t^* and *Tlr7^t/y^* (male) or *Tlr7^t/t^* (female) mice (where *t* indicates the dual Myc-HA tag).

Next, we examined the expression and distribution of TLR3 and TLR7 in our Myc-HA-tagged knockin mouse lines using anti-HA and anti-Myc tag antibodies. Hereafter, we denote these dual-tagged TLR3 and TLR7 proteins as TLR3-MH and TLR7-MH, respectively. We tested commercially available anti-HA tag antibodies—namely rabbit monoclonal C29F4, rat monoclonal 3F10 and mouse monoclonal 16B12 antibodies—as well as one anti-Myc tag antibody (mouse monoclonal 9B11), to detect these recombinant proteins (**Supplementary Table 2**). Among them, anti-HA antibody 3F10 performed best in detecting TLR3-MH or TLR7-MH in immunoblotting and immunoprecipitation assays (**Figure 3** and **Supplementary Table 2**), despite some non-specific bands appearing in immunoblots of total lysates from brain and heart (**Figure 3B**). Anti-HA antibody C29F4 performed well in immunofluorescence staining, immunoprecipitation, and immunoblotting analysis of BMDMs lysates, whereas anti-HA antibody 16B12 was effective in immunofluorescence staining (**Supplementary Table 2**). In contrast, anti-Myc antibody 9B11 proved ineffective at detecting endogenous TLR3-MH as it insufficiently recognized the tagged protein and displayed multiple nonspecific bands in mouse tissue lysates (**Supplementary Figure 6**). Thus, by using specific anti-HA antibodies, our Myc-HA knockin mice are potentially useful for investigating the expression, distribution and complex formation of endogenous TLR3 and TLR7 both *in vivo* and *in vitro*.

### Expression, Cleavage and Glycosylation of TLR3-MH Protein *In Vivo*

Since *Tlr3* mRNA levels in mouse cells/tissues have been revealed previously (31–33), we first examined the expression of TLR3-MH proteins in different organs of *Tlr3^t/t^* mice by immunoblotting, which generated four important results. First, unlike in WT mice, we detected in varying amounts a protein of ~70 kDa using anti-HA antibody in all examined organs of *Tlr3^t/t^* mice (**Figure 3B**). In addition, much lower HA immunoreactivity was observed at ~130 kDa for some *Tlr3^t/t^* organs, such as brain, lung and spleen (**Figure 3B**). Since full-length (FL) TLR3 is cleaved into the NTF and CTF fragments upon transport into endosomes (18, 20), the 70-kDa and 130-kDa proteins present in our *Tlr3^t/t^* mouse tissue lysates likely represent the CTF and FL of TLR3-MH, respectively. Given that amounts of CTF was much higher than for FL TLR3-MH, this result implies that the TLR3-MH proteins are ready to be processed and transported after synthesis *in vivo*.

Second, considering the organs we examined, TLR3-MH displays highest amounts in spleen and lung (**Figure 3B**), which is consistent with TLR3’s function in triggering the innate immune response in immune organs (such as spleen) and organs most exposed to the environment (such as lung). Apart from spleen and lung, we also observed quite high levels of TLR3-MH in brain, liver, kidney and testis (**Figure 3B**). This result evidences different expression levels of TLR3 in various organs.

Third, the ratio of FL to CTF for TLR3-MH varies in different organs. For instance, even though amounts of the CTF fragment of TLR3-MH were comparable between spleen and lung, we noted higher amounts of FL TLR3-MH in the former (**Figure 3B**). Similarly, when we compared levels in brain, liver and kidney, although CTF levels were lower in brain compared to liver and kidney, amounts of FL TLR3-MH were higher in brain than detected in liver and kidney, no matter whether we assayed crude lysates or immunoprecipitated products (**Figure 3C**). These results imply that proteolytic processing of TLR3 varies in different organs.

Finally, the mobility, and thus size, of FL TLR3-MH in SDS-PAGE also varied across tested tissues (**Figures 3B, C, E**), likely due to differential glycosylation. Since glycosylation of the TLR ectodomain is known to be critical for TLR trafficking, complex assembly, responses to ligand stimulation, and signal transduction (34–36), varied glycosylation patterns may influence TLR function in different organs.

We had added the dual Myc-HA tag to the C-terminal end of TLR3, so anti-HA antibody could not be used to monitor expression of the NTF fragment of TLR3-MH. However, we searched the literature and found a published TLR3 antibody, PaT3, which recognizes the N-terminal of TLR3 in immunoprecipitation and immunostaining assays (37) (**Figure 3D**). The PaT3 antibody indeed precipitated FL TLR3-MH from *Tlr3^t/t^* mouse brain lysates (**Figure 3E**). In addition to FL TLR3-MH, we also found that the PaT3 immunoprecipitates contained large amounts of the CTF fragment, in fact >10-fold that of FL TLR3-MH (**Figure 3E**). Since the PaT3 antibody does not work in immunoblotting (37), we were unable to monitor levels of NTF in lysates and immunoprecipitates. Nevertheless, the cleaved NTF and CTF fragments of TLR3 have been shown to still associate with each other in cells for ligand binding and signal transduction (20, 37), so the presence of CTF in PaT3 immunoprecipitates is likely due to interactions between NTF and CTF.

Thus, the analyses using our *Tlr3^t/t^* mice reveal four properties of endogenous TLR3, which were not identified previously. When combined with specific HA antibodies, our *Tlr3^t/t^* mice are powerful tools for investigating expression of endogenous TLR3 in various tissues.

### Expression and Distribution of TLR3-MH in *Tlr3^t/t^* Primary Cultures of BMDMs and Microglial Cells

We examined the expression of TLR3-MH proteins in primary cultures of BMDMs and glial cells from *Tlr3^t/t^* mice and their WT littermates. Similar to our results from mouse tissue lysates, we observed both FL and CTF of TLR3-MH in BMDMs and glial cells using HA antibody. The amounts of the cleaved CTF fragment were still much higher than for FL protein (**Supplementary Figures 7A, B**). We also monitored the expression pattern of TLR3-MH in these primary cultures by immunostaining. Using phalloidin to label F-actin at the cell periphery, we found that TLR3-MH signal presented a punctate pattern in BMDMs (**Supplementary Figure 7C**). In microglia colabeled by IBA1 antibody, HA antibody also revealed a punctate signal of TLR3-MH proteins (**Supplementary Figure 7D**). These results indicate that TLR3-MH is located in intracellular compartments of primary cell cultures prepared from *Tlr3^t/t^* mice.

### Triggering Gene Expression by TLR3-MH Activation

**Figure 4:**
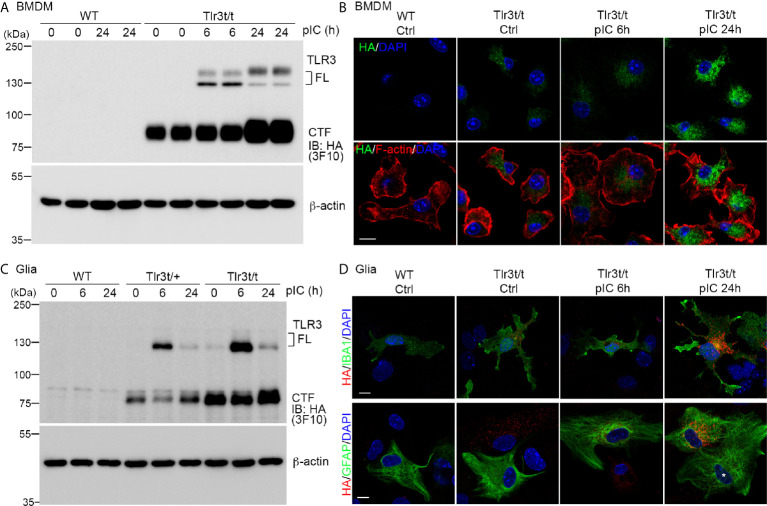
Poly(I:C) stimulation increased endogenous TLR3-MH protein level in *Tlr3^t/t^* cells. **(A, C)** Immunoblot analysis of TLR3 proteins in BMDMs **(A)** and glia cells **(C)**, as indicated. WT, *Tlr3^t/+^* and *Tlr3^t/t^* cells were treated with poly(I:C) or vehicle control. After different incubation times, cell lysates were harvested and analyzed using anti-HA (3F10) and β-actin antibodies (loading control). FL, full-length TLR3; CTF, C-terminal fragment of TLR3. **(B, D)** Immunofluorescence staining to monitor TLR3 expression in BMDMs **(B)** and glia cells **(D)** of WT and *Tlr3t/t* mice with or without poly(I:C) treatment. Anti-HA antibodies (C29F4 in **(B, D)** lower panel, or 16B12 in **(D)** upper panel) were used for dual immunostaining with cell markers. F-actin was used to outline BMDM morphology. IBA1 is a microglia marker. GFAP is a marker for astrocytes. Nuclei were counter-stained with DAPI. Scale bar, 10 μm.

As shown in **Figure 1**, TLR3 activation induces expression of many downstream genes, including *Tlr3* itself, as well as cytokines and chemokines (21, 22, 38). To confirm that TLR3-MH is responsive to a TLR3 ligand, we applied polyinosinic:polycytidylic acid (poly(I:C)), a synthetic dsRNA, to cultured BMDMs and glial cells for 6 and 24 h and monitored TLR3 expression (**Figure 4**). Compared with control groups, poly(I:C) treatment for 6 h noticeably enhanced FL TLR3-MH protein levels (**Figure 4A**). Treatment for 24 h further increased the level of the CTF fragment (**Figure 4A**). TLR3-MH glycosylation levels in BMDMs remarkably differed between the 6- and 24-h treatments (**Figure 4A**). Immunostaining also revealed a robust increase in TLR3-MH levels in BMDMs after 24 h of poly(I:C) stimulation (**Figure 4B**).

In glial cell cultures, we also observed clearly enhanced TLR3-MH expression upon poly(I:C) treatment, but the proteolysis and glycosylation patterns were different from that in poly(I:C)-treated BMDMs (**Figure 4C**). FL TLR3-MH proteins rapidly accumulated 6 h after poly(I:C) treatment and had been cleaved to generate the CTF fragment within 24 h of poly(I:C) stimulation (**Figure 4C**). Fluorescence staining also indicated strong expression of TLR3-MH proteins in both microglia and astrocytes 24 h after poly(I:C) treatment (**Figure 4D**). We noticed that some GFAP-positive astrocytes presented a strong response to poly(I:C), resulting in high-level expression of TLR3-MH (**Figure 4D**), whereas others displayed a low level response, even after 24 h of treatment (**Figure 4D**, white asterisk). This outcome indicates that there were at least two different types of astrocytes in our glial cell cultures in terms of responsiveness to poly(I:C) stimulation.

**Figure 5:**
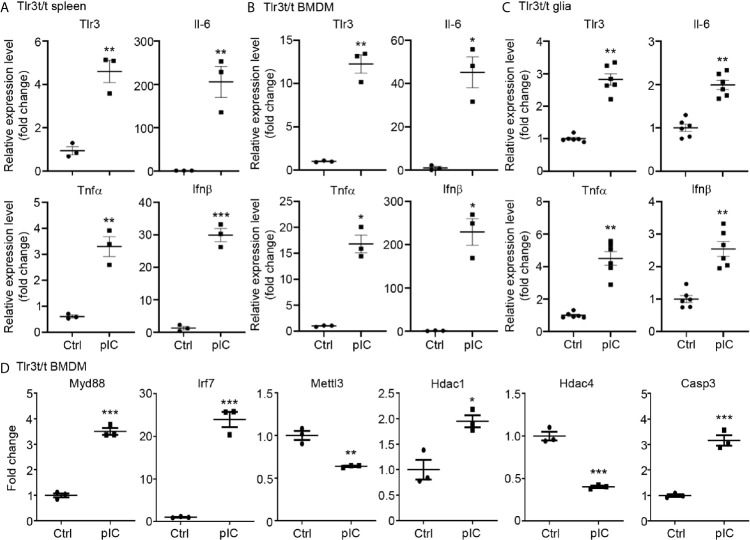
Poly(I:C) stimulation altered the expression of *Tlr3* and downstream genes in *Tlr3^t/t^* spleen and cells. **(A)** Quantitative RT-PCR analysis of *Tlr3* and the cytokines *Il-6*, *Tnfα* and *Ifnβ* in poly(I:C)-challenged *Tlr3^t/t^* mouse spleen. Six hours after an intraperitoneal injection of 5 mg/kg poly(I:C), total RNA was extracted from spleens of *Tlr3^t/t^* mice using TRIzol reagent. The expression levels of indicated cytokine genes were normalized against the internal control *Hprt*. **(B–D)** Primary cultures of BMDMs **(B, D)** and glia cells **(C)** from *Tlr3^t/t^* mice were stimulated with 10 μg/ml poly(I:C) for 6 h. The relative RNA levels of indicated genes normalized against the internal control *Hprt* were analyzed by means of quantitative RT-PCR. Data are represented as mean ± SEM (*error bars*). Each dot indicates the result of **(A)** an individual animal or **(B–D)** an independent culture. *p > 0.05; **p > 0.01; ***p > 0.001.

We further employed quantitative RT-PCR to investigate the response of TLR3-MH to poly(I:C) stimulation. First, we intraperitoneally injected poly(I:C) or vehicle control into *Tlr3^t/t^* mice. Six hours later, spleens were harvested for analysis. Compared with vehicle control, poly(I:C) treatment significantly increased levels in *Tlr3^t/t^* mice of *Tlr3* RNA as well as of different cytokines, including *Il-6*, *Tnfα* and *Ifnβ* (**Figure 5A**). In cultured BMDMs and glial cells, poly(I:C) treatment for 6 h also enhanced transcript levels of *Tlr3*, *Il-6*, *Tnfα* and *Ifnβ* (**Figures 5B, C**). We further investigated whether TLR3-MH activation also regulates expression of TLR3 target genes identified from transcriptomic profiling in WT cells (**Figures 1**, **2**). Consistent with the upregulation of TLR3 and cytokines, TLR3-MH activation also increased expression of *Myd88, Irf7, Hdac1* and *Casp3* but reduced the levels of *Mettl3* and *Hdac4* (**Figure 5D**). These results indicate that TLR3-MH is functional in terms of delivering its immune signal and inducing gene expression, including of *Tlr3* itself and downstream cytokines. Thus, our *Tlr3^t/t^* mice can be deployed to track TLR3 expression and function *in vivo* and *in vitro*.

### Failure of Inducing Cytokine Expression by TLR7-MH Activation

**Figure 6:**
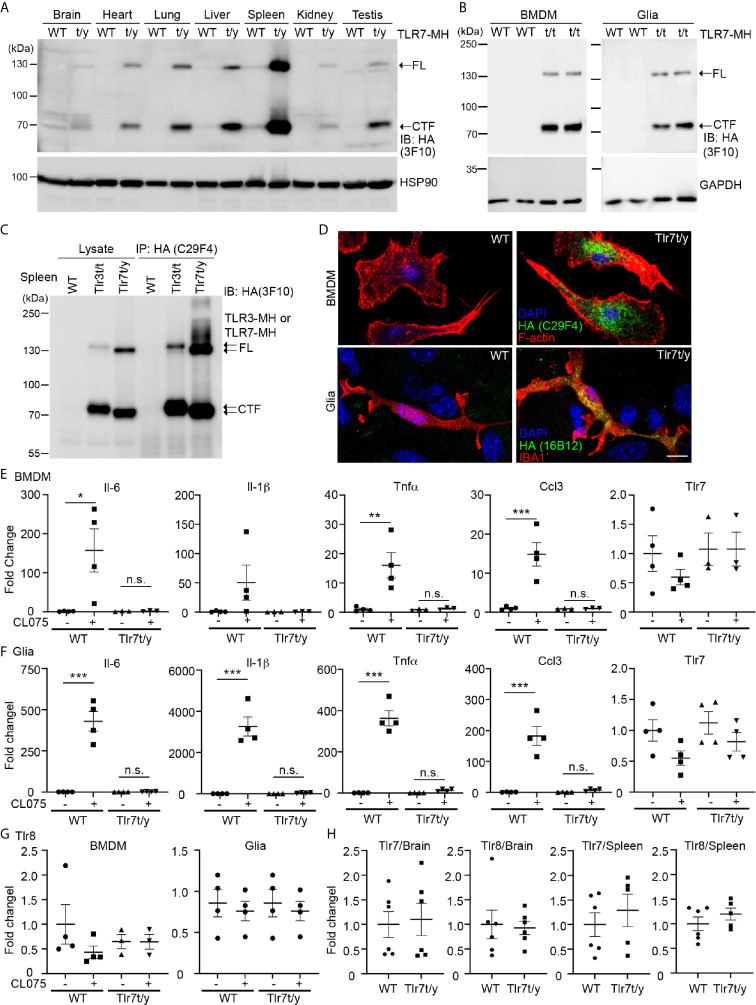
C-terminal Myc-HA dual tagging results in inactivation of TLR7. **(A, B)** Immunoblotting using anti-HA antibodies was performed to detect the expression of TLR7-MH in *Tlr7^t/y^* tissues **(A)** and *Tlr7^t/t^* cells **(B)**. Different organs and BMDMs and glia cell cultures were examined, as indicated. Samples prepared from WT littermates were used as negative controls. HSP90 and GAPDH were used as a loading control. FL, full-length TLR7-MH; CTF, C-terminal fragment of TLR7-MH. **(C)** Distinct levels of proteolyzed TLR3-MH and TLR7-MH in mouse spleen. Anti-HA antibody (C29F4) was used to precipitate TLR3-MH and TLR7-MH proteins from *Tlr3^t/t^* and *Tlr7^t/y^* mouse spleens, respectively. WT mice were used as a negative control. The IP complex was then subjected to IB analysis using anti-HA antibody (3F10). FL: full-length TLR3-MH or TLR7-MH, CTF: C-terminal fragment of TLR3-MH or TLR7-MH. ** (D) ** Immunostaining of BMDMs and glial cells using anti-HA and anti-IBA1 (for microglia) antibodies was performed, as indicated. Phalloidin and DAPI were used to label F-actin and nuclei, respectively. Scale bars, 10 μm. **(E–G)** BMDMs and glia cells prepared from WT and *Tlr7^t/t^* mice were treated with 4 μM CL075, a TLR7 agonist, for 6 h. Expression levels of *Il-6*, *Il-1b*, *Tnfa*, *Ccl3*, *Tlr7* and *Tlr8* were then determined using quantitative RT-PCR and normalized against the internal control *Hprt*. **(H)** Levels of *Tlr7* and *Tlr8* mRNA are similar between WT and *Tlr7^t/y^* mouse organs. Brain and spleen of WT and *Tlr7^t/y^* mice were harvested for RNA extraction and then subjected to quantitative RT-PCR to analyze levels of *Tlr7* and *Tlr8*, normalized against the internal control *Hprt*. Data are represented as mean ± SEM (*error bars*). Each dot indicates the result of one independent culture. *p > 0.05; **p > 0.01; ***p > 0.001; n.s., non-significant. **(E–G)** Two-way with Bonferroni’s multiple comparisons test.

We then applied the same approaches to characterize *Tlr7^t/y^* (male) and/or *Tlr7^t/t^* (female) mice. Similar to our observations for TLR3-MH, we detected FL and the cleaved CTF fragment of TLR7-MH proteins in different tissues and BMDM and glial cell primary cultures *via* immunoblot analyses (**Figures 6A, B**). However, unlike TLR3-MH, TLR7-MH was predominantly expressed in spleen rather than in the other organs we examined (**Figure 6A**). Comparing TLR3-MH and TLR7-MH proteins in *Tlr3^t/t^* and *Tlr7^t/y^* spleen lysates, we noted that amounts of the CTF fragments of TLR3-MH and TLR7-MH were similar, whereas levels of FL TLR7-MH were much higher than for FL TLR3-MH in both lysates and immunoprecipitates (**Figure 6C**). These results indicate that proteolytic processing of TLR3 and TLR7 could differ.

Immunofluorescence staining also revealed an intracellular punctate pattern of TLR7-MH in BMDMs and glial cells (**Figure 6D**), which is similar to the staining pattern observed for TLR3-MH (**Figures 4B, D** and **Supplementary Figures 7C, D**). However, unlike TLR3-MH, TLR7-MH did not respond to its agonist CL075, a thiazoloquinolone derivative (12, 22). To activate mouse TLR7, we added CL075 into primary cultures of BMDMs or glial cells for 6 h, but found that it did not induce expression of *Il-6*, *Tnfα*, *Ccl3*, or *Il-1β* in *Tlr7^t/y^* cells (**Figures 6E, F**). In contrast to *Tlr7^t/y^* cells, CL075 effectively triggered expression of the *Il-6*, *Tnfα*, *Il-1β* and *Ccl3* genes in WT cells (**Figures 6E, F**). These results indicate that although TLR7-MH can be proteolytically processed into the NTF and CTF fragments, it lacks the ability to trigger downstream signaling and to subsequently induce cytokine expression.

Notably, unlike TLR3 (**Figures 1**, **5**), TLR7 activation did not elicit a positive feedback mechanism to induce its own expression in either BMDMs or glial cells prepared from WT mice (**Figures 6E, F**), suggesting that the downstream signaling and transcriptional regulatory mechanisms of TLR3 and TLR7 differ. Our previous studies have shown that *Tlr7* knockout upregulates *Tlr8* expression in brain or cultured neurons (12, 22). Although TLR7-MH appears not to be functional in *Tlr7^t/y^* mice, we found that *Tlr8* transcripts were not increased in primary cell cultures or in brain/spleen tissues (**Figures 6G, H**). Since TLR7-MH signaling is blocked and TLR8 is not upregulated in *Tlr7^t/y^* mice, they may serve as a more precise model for functional study of TLR7.

In conclusion, our results have shown that applying the same tagged knockin approach results in different outcomes for TLR3 and TLR7, with TLR3-MH remaining functional whereas TLR7-MH loses the ability to induce an immune response.

### TLR7-MH Does Not Form Signalosomes With MYD-88

**Figure 7:**
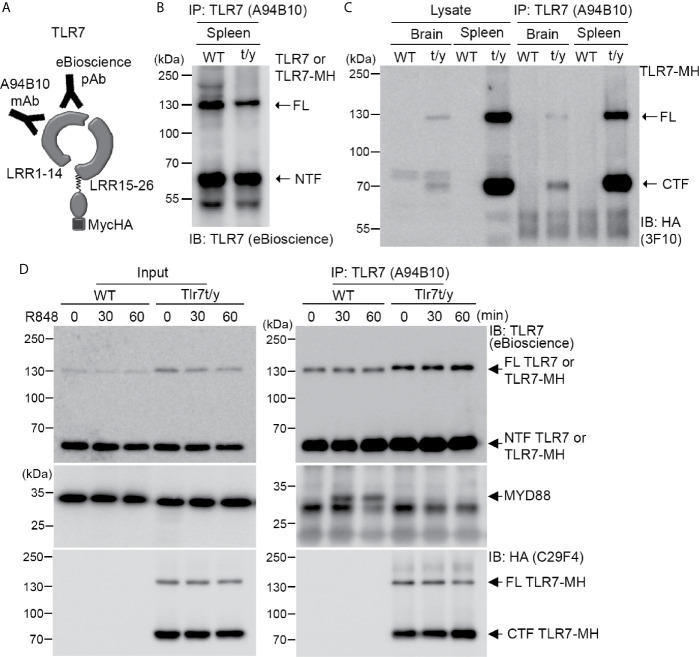
Dual C-terminal Myc-HA tagging disrupts the interaction between TLR7 and MYD88. **(A)** Schematic of TLR7 protein with the dual C-terminal Myc-HA tag. Monoclonal anti-TLR7 antibody (A94B10) and polyclonal TLR7 antibody both target the N-terminal region. **(B, C)** The N-terminal (NTF) and C-terminal (CTF) fragments of TLR7 remain associated with each other after proteolytic cleavage. TLR7 was immunoprecipitated (IP) using anti-TLR7 antibody (A94B10) from brain and spleen lysates of WT and *Tlr7^t/y^* mice. The IP complex was then subjected to immunoblotting (IB) analysis using anti-TLR7 antibody (eBioscience) **(B)** or anti-HA antibody (3F10) **(C)**. **(D)** WT and *Tlr7^t/y^* BMDMs were treated with a TLR7 agonist (R848) for 30 and 60 min. Cell lysates were then collected and subjected to IP using anti-TLR7 antibody (A94B10). The IP complex was then analyzed by IB using anti-TLR7 (eBioscience) (upper panel), anti-MyD88 (middle panel), and anti-HA (C29F4, lower panel) antibodies. FL, full-length.

Next, we investigated how our C-terminal Myc-HA tagging of TLR7 impaired its activity. As for other endosomal TLRs, the NTF and CTF fragments of TLR7 remain linked to each other after cleavage and are required for TLR7 responses (17, 19). Accordingly, we investigated if the NTF and CTF of TLR7-MH still interact with each other. We employed monoclonal TLR7 antibody (A94B10) (19) and polyclonal TLR7 antibody (eBioscience) that both recognize the N-terminal region of TLR7 (**Figure 7A**) for immunoprecipitation and immunoblotting, respectively, allowing us to monitor TLR7 or TLR7-MH protein complexes *in vivo*. Our data show that A94B19 antibody precipitated both the FL and NTF of TLR7 and TLR7-MH from WT and *Tlr7^t/y^* mouse spleen lysates, respectively (**Figure 7B**). Similar to our observations for TLR3, we detected the cleaved CTF fragment of TLR7-MH by anti-HA antibody in the immunoprecipitated complex (**Figure 7C**), suggesting that the NTF and CTF of TLR7-MH likely interact with each other.

MYD88 is a critical signaling adaptor molecule for most TLRs, including TLR7 (1, 4, 39). Upon ligand binding, the cytoplasmic Tir domain of TLRs associates with MYD88 to recruit other downstream molecules, allowing the resulting signalosome to trigger cytokine expression (1, 4, 40). To test if C-terminal Myc-HA tagging influenced MYD88 binding, we treated WT and *Tlr7^t/y^* BMDM primary cultures with a TLR7 ligand, R848, for 30 and 60 min. The cell lysates were then subjected to immunoprecipitation and immunoblotting analyses using TLR7 (A94B10) and MYD88 antibodies. We found that only WT TLR7 coimmunoprecipitated with MYD88 (**Figure 7D** middle panel). In contrast, MYD88 was not present in TLR7-MH complexes, even though amounts of FL and CTF of TLR7-MH were higher than for WT TLR7 (**Figure 7D**). Similar to our observations of spleen and brain lysates, the NTF and CTF fragments of TLR7-MH still remained associated in BMDM primary cultures (**Figure 7D**). Thus, adding the Myc-HA tag at the C-terminal end of TLR7 disrupts signalosome complex formation, thereby impairing TLR7 responses.

## Discussion

In this report, we demonstrated the different properties of TLR3 and TLR7 in mouse tissues and primary cultured cells. First of all, transcriptomic profiling indicates that TLR3 and TLR7 regulate expression of distinct gene sets in BMDMs. We found that TLR3 activation generally increased the expression of all *Tlrs* (except *Tlr5*), Tir domain-containing adaptors (except *Sarm1*), and inflammation-related transcriptional factors (except *Irf3*) (**Figure 1C**). In contrast, TLR7 activation generally downregulated the expression of *Tlr* genes, except for *Tlr1*, *Tlr2* and *Tlr6* (**Figure 1C**). Thus, TLR3 activation globally upregulates the innate immune responses of BMDMs. However, TLR7 activation likely attenuates or restricts innate immunity mediated by other TLRs. It remains unclear how TLR7 activation specifically upregulates the expression of *Tlr1*, *Tlr2* and *Tlr6*. These three *Tlr* genes all belong to the *Tlr1* subfamily and are believed to have evolved *via* a series of independent gene duplications and subsequently diverged by positive selection (4, 41). Thus, it is likely that the promoter and enhancer regions of these genes are all conserved and controlled by similar transcription factors. Since the TLR1 subfamily recognizes lipopeptides derived from microbes, it would also be interesting to explore the physiological relevance of enhanced lipopeptide recognition upon TLR7 activation.

In addition to TLRs, we also noticed that TLR3 activation greatly increased expression of *Myd88* (~4-fold) but not *Trif*. For TLR7 activation, both *Myd88* and *Trif* were increased around 1 to 2 fold (**Figure 2**). These results were relatively unexpected as TLR3 was auto-upregulated by TLR3 activation and TRIF is known to be the critical downstream adaptor of TLR3 to induce cytokine expression. It is unclear whether the existing TRIF is sufficient for overexpressed TLR3 to deliver the signaling. For TLR7 activation, it did not induce *Tlr3* expression but increased *Trif* expression (**Figure 2**). It is interesting to further investigate whether TRIF has TLR3-independent function, such as regulating other TLRs’ signaling (4).

Furthermore, we generated two knockin mouse lines displaying dual Myc-HA tagging at the C-terminal ends of the *Tlr3* and *Tlr7* genes. Using commercially-available anti-HA antibodies, we were able to establish specific expression profiles for endogenous TLR3 and TLR7 *in vivo* and also to visualize the subcellular distributions of TLR3 and TLR7 in different cell types. Moreover, we used anti-HA antibody to compare endogenous TLR3 and TLR7 proteins. Our data shows that TLR7 proteins are predominantly expressed in spleen, though lower amounts of that protein are still present in other organs. Apart from noticeably high levels of TLR3 being expressed in spleen and lung, considerable amounts were detected in most of the organs we examined. Although levels of cleaved fragments of both TLR3 and TLR7 were higher relative to uncleaved FL proteins, relative amounts of FL TLR7 are still greater than those of FL TLR3. In addition, proteolytic processing efficiency and posttranslational modification of TLR7 are comparable among different organs. In contrast, we found that TLR3 displayed differential posttranslational modification and proteolytic processing efficiency in different tissues. Our *Tlr3^t/t^* and *Tlr7^t/y^* mice enable investigation of the differences between TLR3 and TLR7 and facilitate assessments of endogenous TLR3 and TLR7 under more physiologically relevant conditions.

Our C-terminal Myc-HA tagging had different consequences for TLR3 and TLR7. The TLR3-MH protein still responded to poly(I:C), which triggered subsequent expression of cytokines and chemokines. Thus, TLR3-MH is a functional protein and our *Tlr3^t/t^* mice can be used for all kinds of TLR3-related studies. Unexpectedly, the same C-terminal Myc-HA tag disrupted the association of TLR7-MH with MYD88 and, upon ligand stimulation, downstream signaling was impaired. The Myc-HA-tagged *Tlr7* mice can be considered a TLR7 loss-of-function animal model in which gene expression of nearby genes, such as *Tlr8*, is unaffected. It will be interesting to compare if there is any difference between *Tlr7*-knockout and Myc-HA tagged mice. It would also be intriguing to investigate how the extreme C-terminal tail of TLR7 contributes to MYD88 interaction.

Previous studies have generated reporter mice for *Tlr2*, *Tlr4*, *Tlr5*, *Tlr7* and *Tlr9* with a view to assessing TLR expression (42, 43). In those cases, either a HA or Flag tag was placed at the 3’ end of the respective *Tlr* genes, followed by an IRES sequence and various fluorescence proteins. The fluorescence proteins labeled TLR-expressing cells and the C-terminal HA or Flag tag could be used to detect the TLR proteins (42, 43). In our study, we found that C-terminal tagging with a Myc-HA cassette impaired signaling by TLR7 but not by TLR3. Thus, we feel it is worth reinvestigating the aforementioned published reporter mouse lines to establish if the tagged TLRs respond normally to their ligands. It will also be interesting to compare the sensitivity of different TLRs to C-terminal tagging. If as for TLR7, other TLRs are sensitive to C-terminal tagging and lose their ability to deliver downstream signaling, it suggests that they may all form similar signalosomes for signal transduction. If contrasting results arise from such an experiment, it would suggest that the extreme C-terminal ends of different TLRs may contribute differentially to signal transduction.

Both TLR7 and TLR8 recognize ssRNA and regulate neuronal morphology and function (12, 22). Our previous study showed that *Tlr7* knockout upregulates *Tlr8* expression in brain (12, 22). This compensatory effect is brain-specific, and is not observed in spleen (12, 22). Here, we report that TLR7-MH lacks the ability to deliver downstream signaling. Thus, our *Tlr7^t/y^* mice still represent *Tlr7* knockout mice even though they still express TLR7-MH protein. Interestingly, we found that *Tlr8* transcripts were not increased in *Tlr7^t/y^* mice, no matter whether we assessed BMDMs, glial cells or tissues. Although *Tlr7^–/y^* knockout mice exhibit some behavioral abnormalities in terms of olfaction, anxiety, aggression and contextual fear memory (25), we cannot be sure if *Tlr8* upregulation also influences the phenotype of *Tlr7* knockout mice. Compared with *Tlr7^–/y^* mice, the *Tlr7^t/y^* mouse line we report herein may represent a good or even better genetic model for *Tlr7* because it minimizes the potential interference contributed by *Tlr8* upregulation. Investigations of the behavioral characteristics of *Tlr7^t/y^* mice are warranted.

The proteolytic processing of TLRs has been studied for both endogenous proteins and in overexpression systems. For endogenous proteins, the proteolytic products of endogenous TLR3, TLR7, and TLR9 are much more abundant than the respective full length proteins in human retinal epithelial cell line RPE1, primary human monocyte-derived dendritic cells (MDDCs), and mouse BMDMs (18, 37, 43, 44), which is consistent with our findings presented herein. In contrast, amounts of proteolyzed TLRs were found to be < 5% relative to FL TLRs in TLR-overexpressing HEK293 cells (19, 20, 45). Using our dual Myc-HA-tagged mouse lines, it is now relatively easy to prepare primary cell cultures for the study of TLR3 and TLR7 under more physiologically relevant conditions, greatly enabling investigations of TLR3 and TLR7 in the future.

## Data Availability Statement

Data is available at https://www.ncbi.nlm.nih.gov/geo/query/acc.cgi?acc=GSE163347.

## Ethics Statement

The animal study was reviewed and approved by Academia Sinica Institutional Animal Care and Utilization Committee.

## Author Contributions

Conceptualization, C-YC, T-FW and M-ZL and Y-PH. Methodology and investigation, C-YC, Y-FH, C-YT, Y-CS, T-FC, T-FW and M-ZL. Writing, C-YC, Y-FH, and Y-PH. Funding acquisition, C-YT and Y-PH. Supervision and project administration, Y-PH. All authors contributed to the article and approved the submitted version.

## Funding

This work was supported by grants from Academia Sinica (AS-IA-106-L04 and AS-TP-110-L10 to Y-PH; AS-CFII-108-104 to C-YT).

## Conflict of Interest

The authors declare that the research was conducted in the absence of any commercial or financial relationships that could be construed as a potential conflict of interest.

## References

[1] Akira S Uematsu S Takeuchi O. Pathogen Recognition and Innate Immunity. Cell (2006) 124:783–801. 10.1016/j.cell.2006.02.015 16497588

[2] Brubaker SW Bonham KS Zanoni I Kagan JC. Innate Immune Pattern Recognition: A Cell Biological Perspective. Annu Rev Immunol (2015) 33:257–90. 10.1146/annurev-immunol-032414-112240 PMC5146691 25581309

[3] Gay NJ Gangloff M. Structure and Function of Toll Receptors and Their Ligands. Annu Rev Biochem (2007) 76:141–65. 10.1146/annurev.biochem.76.060305.151318 17362201

[4] Chen CY Shih YC Hung YF Hsueh YP. Beyond Defense: Regulation of Neuronal Morphogenesis and Brain Functions *Via* Toll-like Receptors. J BioMed Sci (2019) 26:90. 10.1186/s12929-019-0584-z 31684953 PMC6827257

[5] Kawai T Akira S. The Role of Pattern-Recognition Receptors in Innate Immunity: Update on Toll-Like Receptors. Nat Immunol (2010) 11:373–84. 10.1038/ni.1863 20404851

[6] Majer O Liu B Barton GM. Nucleic Acid-Sensing TLRs: Trafficking and Regulation. Curr Opin Immunol (2017) 44:26–33. 10.1016/j.coi.2016.10.003 27907816 PMC5446938

[7] Miyake K Shibata T Ohto U Shimizu T Saitoh SI Fukui R. Mechanisms Controlling Nucleic Acid-Sensing Toll-like Receptors. Int Immunol (2018) 30:43–51. 10.1093/intimm/dxy016 29452403

[8] Diebold SS Kaisho T Hemmi H Akira S Reis e Sousa C. Innate Antiviral Responses by Means of TLR7-mediated Recognition of Single-Stranded RNA. Science (2004) 303:1529–31. 10.1126/science.1093616 14976261

[9] Heil F Hemmi H Hochrein H Ampenberger F Kirschning C Akira S. Species-Specific Recognition of Single-Stranded RNA *Via* Toll-Like Receptor 7 and 8. Science (2004) 303:1526–9. 10.1126/science.1093620 14976262

[10] Alexopoulou L Holt AC Medzhitov R Flavell RA. Recognition of Double-Stranded RNA and Activation of NF-kappaB by Toll-like Receptor 3. Nature (2001) 413:732–8. 10.1038/35099560 11607032

[11] Hemmi H Takeuchi O Kawai T Kaisho T Sato S Sanjo H. A Toll-Like Receptor Recognizes Bacterial DNA. Nature (2000) 408:740–5. 10.1038/35047123 11130078

[12] Liu HY Hong YF Huang CM Chen CY Huang TN Hsueh YP. TLR7 Negatively Regulates Dendrite Outgrowth Through the Myd88-c-Fos-IL-6 Pathway. J Neurosci (2013) 33:11479–93. 10.1523/JNEUROSCI.5566-12.2013 PMC6618696 23843519

[13] Liu HY Chen CY Hsueh YP. Innate Immune Responses Regulate Morphogenesis and Degeneration: Roles of Toll-Like Receptors and Sarm1 in Neurons. Neurosci Bull (2014) 30:645–54. 10.1007/s12264-014-1445-5 PMC5562625 24993772

[14] Liu HY Huang CM Hung YF Hsueh YP. The Micrornas Let7c and miR21 Are Recognized by Neuronal Toll-Like Receptor 7 to Restrict Dendritic Growth of Neurons. Exp Neurol (2015) 269:202–12. 10.1016/j.expneurol.2015.04.011 25917529

[15] Miyake K Saitoh SI Sato R Shibata T Fukui R Murakami Y. Endolysosomal Compartments as Platforms for Orchestrating Innate Immune and Metabolic Sensors. J Leukoc Biol (2019) 106:853–62. 10.1002/JLB.MR0119-020R 31219657

[16] Kim YM Brinkmann MM Paquet ME Ploegh HL. UNC93B1 Delivers Nucleotide-Sensing Toll-Like Receptors to Endolysosomes. Nature (2008) 452:234–8. 10.1038/nature06726 18305481

[17] Ewald SE Engel A Lee J Wang M Bogyo M Barton GM. Nucleic Acid Recognition by Toll-like Receptors is Coupled to Stepwise Processing by Cathepsins and Asparagine Endopeptidase. J Exp Med (2011) 208:643–51. 10.1084/jem.20100682 PMC3135342 21402738

[18] Garcia-Cattaneo A Gobert FX Muller M Toscano F Flores M Lescure A. Cleavage of Toll-like Receptor 3 by Cathepsins B and H is Essential for Signaling. Proc Natl Acad Sci U S A (2012) 109:9053–8. 10.1073/pnas.1115091109 PMC3384196 22611194

[19] Kanno A Yamamoto C Onji M Fukui R Saitoh S Motoi Y. Essential Role for Toll-like Receptor 7 (TLR7)-Unique Cysteines in an Intramolecular Disulfide Bond, Proteolytic Cleavage and RNA Sensing. Int Immunol (2013) 25:413–22. 10.1093/intimm/dxt007 23446849

[20] Toscano F Estornes Y Virard F Garcia-Cattaneo A Pierrot A Vanbervliet B. Cleaved/Associated TLR3 Represents the Primary Form of the Signaling Receptor. J Immunol (2013) 190:764–73. 10.4049/jimmunol.1202173 23255358

[21] Chen CY Liu HY Hsueh YP. TLR3 Downregulates Expression of Schizophrenia Gene Disc1 *Via* MYD88 to Control Neuronal Morphology. EMBO Rep (2017) 18:169–83. 10.15252/embr.201642586 PMC5210159 27979975

[22] Hung YF Chen CY Shih YC Liu HY Huang CM Hsueh YP. Endosomal TLR3, TLR7, and TLR8 Control Neuronal Morphology Through Different Transcriptional Programs. J Cell Biol (2018) 217:2727–42. 10.1083/jcb.201712113 PMC6080926 29777026

[23] Lund JM Alexopoulou L Sato A Karow M Adams NC Gale NW. Recognition of Single-Stranded RNA Viruses by Toll-Like Receptor 7. Proc Natl Acad Sci U S A (2004) 101:5598–603. 10.1073/pnas.0400937101 PMC397437 15034168

[24] Shih PY Hsieh BY Lin MH Huang TN Tsai CY Pong WL. Cttnbp2 Controls Synaptic Expression of Zinc-Related Autism-Associated Proteins and Regulates Synapse Formation and Autism-Like Behaviors. Cell Rep (2020) 31:107700. 10.1016/j.celrep.2020.107700 32492416

[25] Hung YF Chen CY Li WC Wang TF Hsueh YP. Tlr7 Deletion Alters Expression Profiles of Genes Related to Neural Function and Regulates Mouse Behaviors and Contextual Memory. Brain Behav Immun (2018) 72:101–13. 10.1016/j.bbi.2018.06.006 29885943

[26] Liu HY Chen CY Hung YF Lin HR Chao HW Shih PY. Rnase A Promotes Proliferation of Neuronal Progenitor Cells *Via* an ERK-Dependent Pathway. Front Mol Neurosci (2018) 11:428. 10.3389/fnmol.2018.00428 30534052 PMC6275325

[27] Chen CY Lin CW Chang CY Jiang ST Hsueh YP. Sarm1, a Negative Regulator of Innate Immunity, Interacts With Syndecan-2 and Regulates Neuronal Morphology. J Cell Biol (2011) 193:769–84. 10.1083/jcb.201008050 PMC3166868 21555464

[28] Carty M Bowie AG. Recent Insights Into the Role of Toll-like Receptors in Viral Infection. Clin Exp Immunol (2010) 161:397–406. 10.1111/j.1365-2249.2010.04196.x 20560984 PMC2962956

[29] Lester SN Li K. Toll-Like Receptors in Antiviral Innate Immunity. J Mol Biol (2014) 426:1246–64. 10.1016/j.jmb.2013.11.024 PMC3943763 24316048

[30] Kominsky DJ Campbell EL Colgan SP. Metabolic Shifts in Immunity and Inflammation. J Immunol (2010) 184:4062–8. 10.4049/jimmunol.0903002 PMC4077461 20368286

[31] Applequist SE Wallin RP Ljunggren HG. Variable Expression of Toll-like Receptor in Murine Innate and Adaptive Immune Cell Lines. Int Immunol (2002) 14:1065–74. 10.1093/intimm/dxf069 12202403

[32] Singh MV Cicha MZ Nunez S Meyerholz DK Chapleau MW Abboud FM. Angiotensin II-induced Hypertension and Cardiac Hypertrophy are Differentially Mediated by TLR3- and TLR4-dependent Pathways. Am J Physiol Heart Circ Physiol (2019) 316:H1027–38. 10.1152/ajpheart.00697.2018 PMC6580398 30793936

[33] Shmueli A Shalit T Okun E Shohat-Ophir G. The Toll Pathway in the Central Nervous System of Flies and Mammals. Neuromolecular Med (2018) 20:419–36. 10.1007/s12017-018-8515-9 30276585

[34] Sun J Duffy KE Ranjith-Kumar CT Xiong J Lamb RJ Santos J. Structural and Functional Analyses of the Human Toll-like Receptor 3. Role of Glycosylation. J Biol Chem (2006) 281:11144–51. 10.1074/jbc.M510442200 16533755

[35] Sato R Shibata T Tanaka Y Kato C Yamaguchi K Furukawa Y. Requirement of Glycosylation Machinery in TLR Responses Revealed by CRISPR/Cas9 Screening. Int Immunol (2017) 29:347–55. 10.1093/intimm/dxx044 28992181

[36] Leifer CA Medvedev AE. Molecular Mechanisms of Regulation of Toll-Like Receptor Signaling. J Leukoc Biol (2016) 100:927–41. 10.1189/jlb.2MR0316-117RR PMC5069093 27343013

[37] Murakami Y Fukui R Motoi Y Kanno A Shibata T Tanimura N. Roles of the Cleaved N-terminal TLR3 Fragment and Cell Surface TLR3 in Double-Stranded RNA Sensing. J Immunol (2014) 193:5208–17. 10.4049/jimmunol.1400386 25305318

[38] Li J Ye L Wang X Hu S Ho W. Induction of Interferon-Gamma Contributes to Toll-like Receptor 3-Mediated Herpes Simplex Virus Type 1 Inhibition in Astrocytes. J Neurosci Res (2012) 90:399–406. 10.1002/jnr.22758 22057682 PMC3411314

[39] Hemmi H Kaisho T Takeuchi O Sato S Sanjo H Hoshino K. Small Anti-Viral Compounds Activate Immune Cells *Via* the TLR7 MyD88-dependent Signaling Pathway. Nat Immunol (2002) 3:196–200. 10.1038/ni758 11812998

[40] Medzhitov R Preston-Hurlburt P Kopp E Stadlen A Chen C Ghosh S. MyD88 is an Adaptor Protein in the hToll/IL-1 Receptor Family Signaling Pathways. Mol Cell (1998) 2:253–8. 10.1016/S1097-2765(00)80136-7 9734363

[41] Huang Y Temperley ND Ren L Smith J Li N Burt DW. Molecular Evolution of the Vertebrate TLR1 Gene Family–A Complex History of Gene Duplication, Gene Conversion, Positive Selection and Co-Evolution. BMC Evol Biol (2011) 11:149. 10.1186/1471-2148-11-149 21619680 PMC3125219

[42] Price AE Shamardani K Lugo KA Deguine J Roberts AW Lee BL. A Map of Toll-Like Receptor Expression in the Intestinal Epithelium Reveals Distinct Spatial, Cell Type-Specific, and Temporal Patterns. Immunity (2018) 49:560–75.e566. 10.1016/j.immuni.2018.07.016 30170812 PMC6152941

[43] Roberts AW Lee BL Deguine J John S Shlomchik MJ Barton GM. Tissue-Resident Macrophages Are Locally Programmed for Silent Clearance of Apoptotic Cells. Immunity (2017) 47:913–27.e916. 10.1016/j.immuni.2017.10.006 29150239 PMC5728676

[44] Cavassani KA Ishii M Wen H Schaller MA Lincoln PM Lukacs NW. TLR3 is An Endogenous Sensor of Tissue Necrosis During Acute Inflammatory Events. J Exp Med (2008) 205:2609–21. 10.1084/jem.20081370 PMC2571935 18838547

[45] Qi R Singh D Kao CC. Proteolytic Processing Regulates Toll-Like Receptor 3 Stability and Endosomal Localization. J Biol Chem (2012) 287:32617–29. 10.1074/jbc.M112.387803 PMC3463343 22865861

