# Supplementary material for: Transcriptomic Analysis and C-Terminal Epitope Tagging Reveal Differential Processing and Signaling of Endogenous TLR3 and TLR7
Source: Front Immunol. 2021 Jun 15;12:686060. doi: 10.3389/fimmu.2021.686060 (PMC8240634; doi:10.3389/fimmu.2021.686060)
Supplement: Supplementary file 1 [file DataSheet_1.pdf]

Supplementary Material

Supplementary Figures and Tables

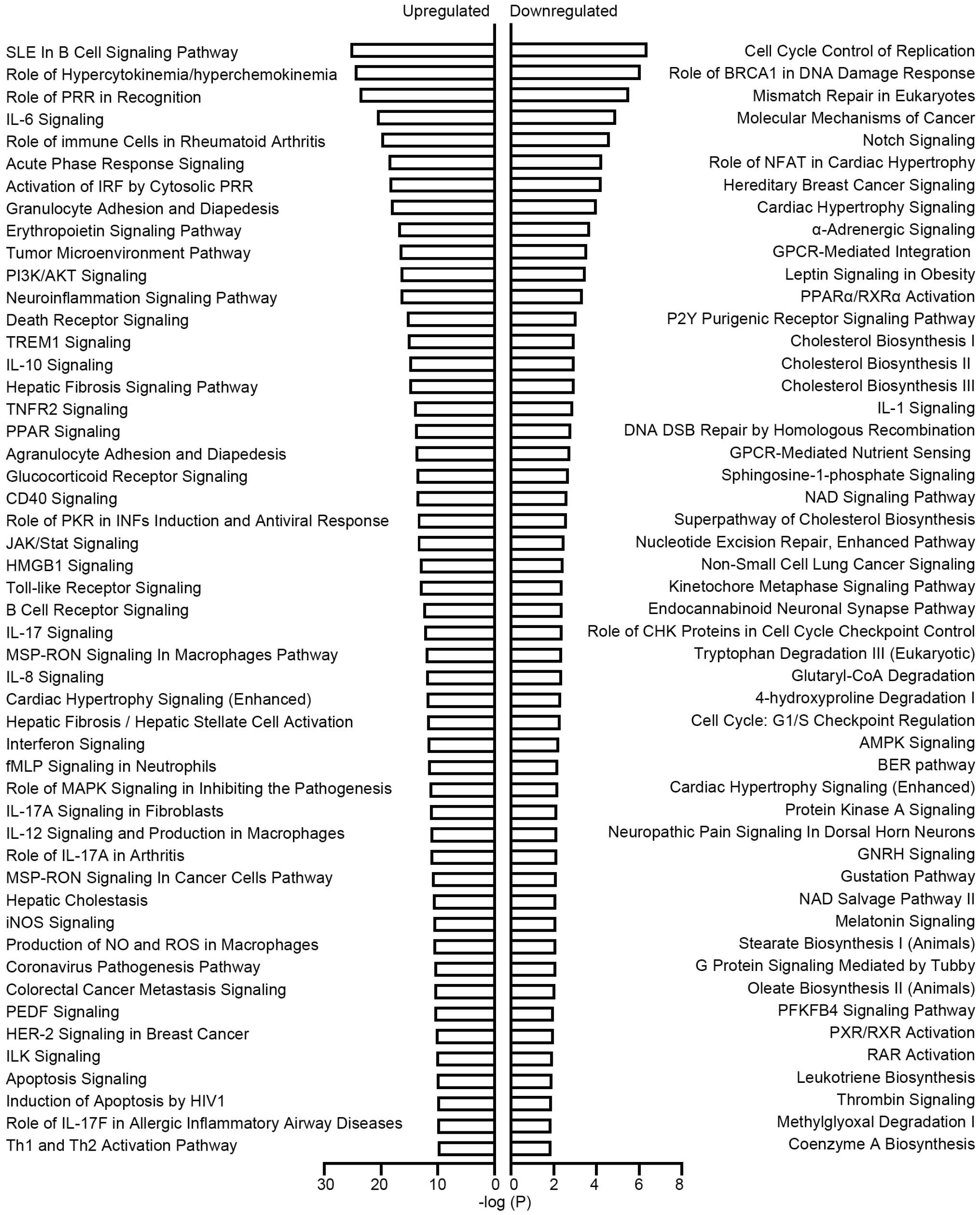

Supplementary Figure 1. Top 50 canonical signaling pathways of shared target genes of TLR3 and TLR7. Ingenuity Pathway Analysis (IPA) was performed to investigate the involvement of shared target genes of TLR3 and TLR7 in various signaling pathways. More than 400 pathways were identified based on criteria of –log(p) > 1 and a ratio > 0.125. Only the top 50 pathways are shown here.

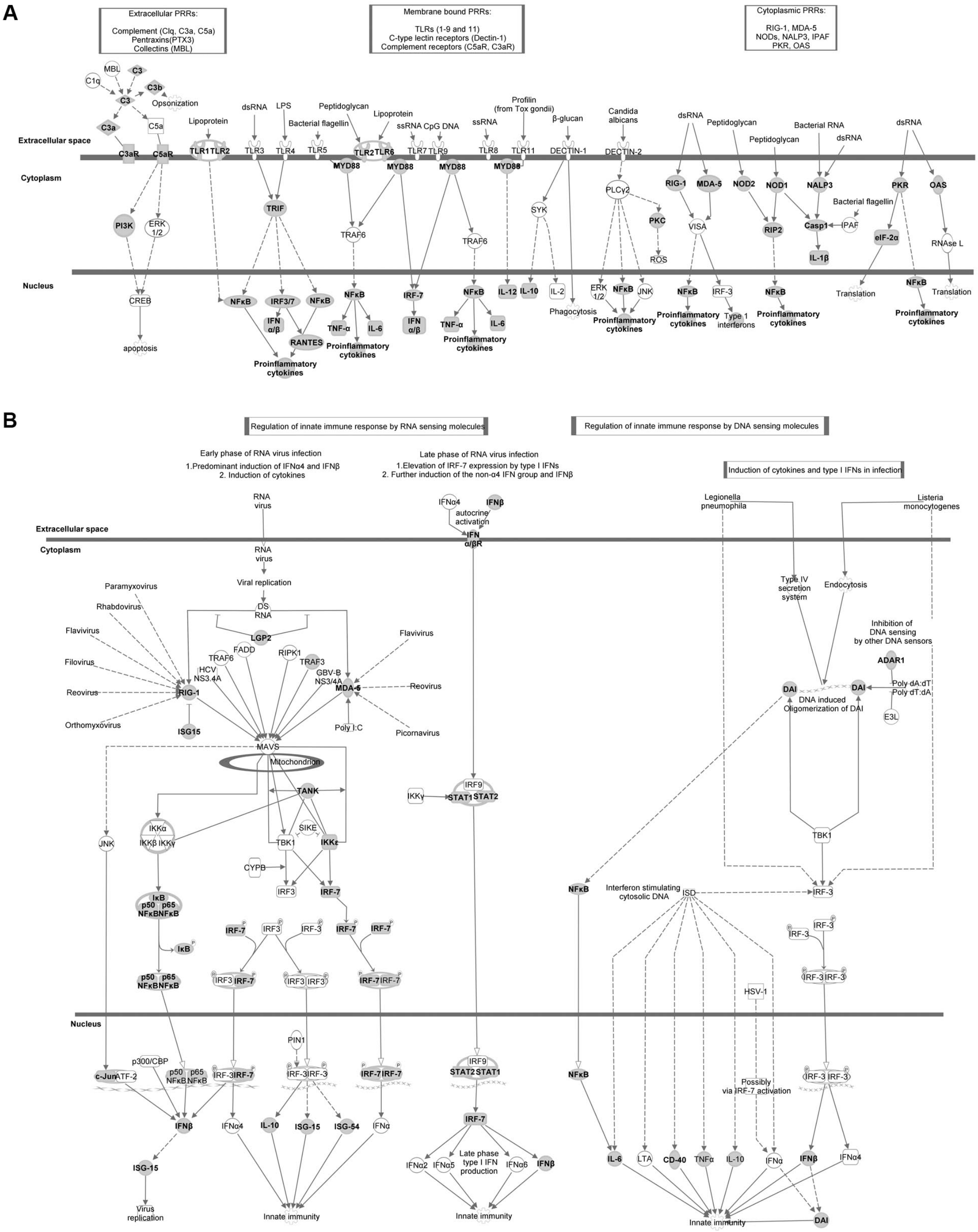

Supplementary Figure 2. Involvement of shared target genes of TLR3 and TLR7 in the pathways of pattern recognition receptors and IRF activation. (A) The role of pattern recognition receptors in recognition of bacteria and viruses. (B) Activation of IRF by cytosolic pattern recognition receptors. Upregulated shared target genes of TLR3 and TLR7 are indicated as grey blocks.

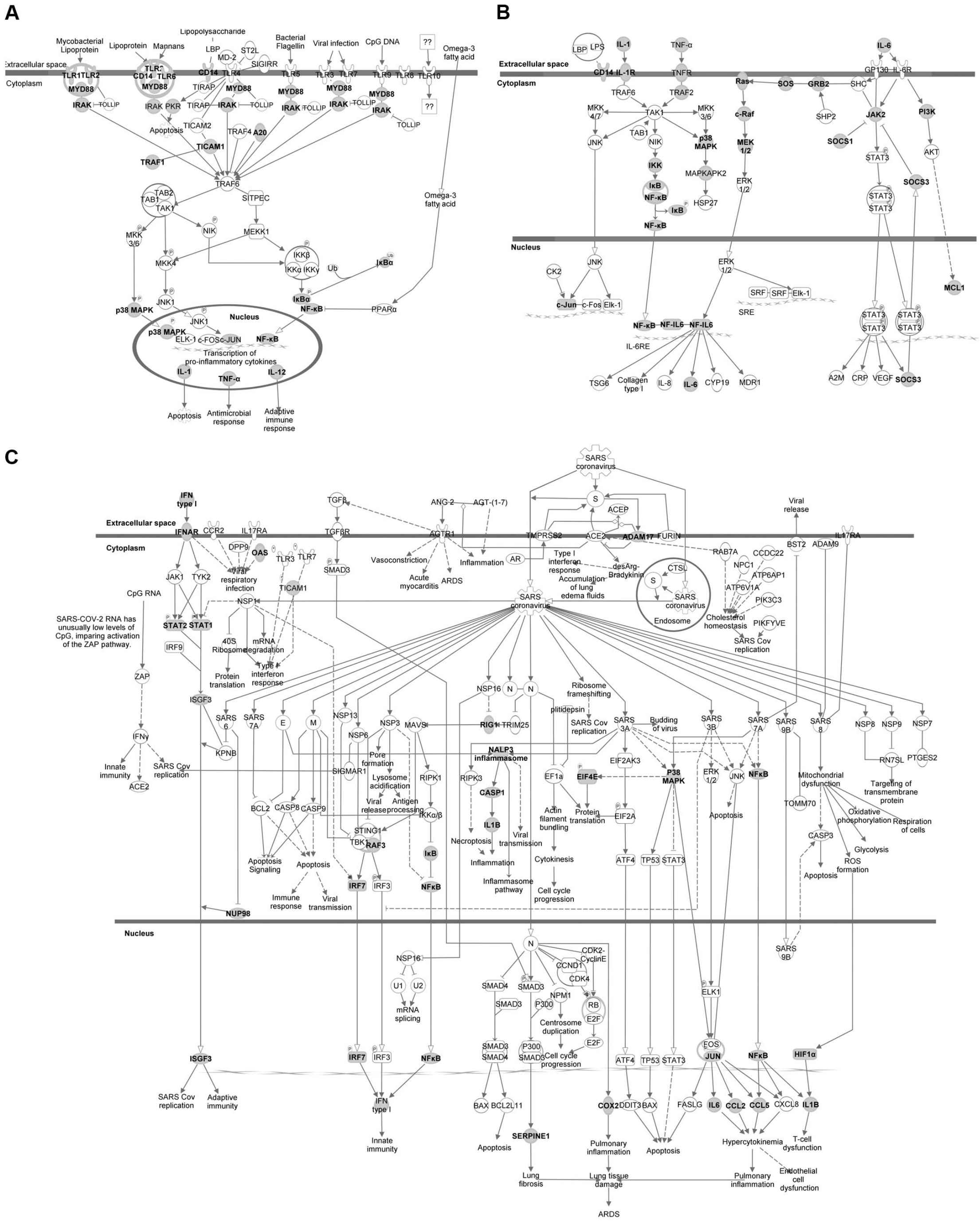

Supplementary Figure 3. Involvement of shared target genes of TLR3 and TLR7 in TLR and IL-6 signaling pathways and the coronavirus pathogenesis pathway. (A) TLR signaling. (B) IL6 signaling. (C) Coronavirus pathogenesis pathway. Upregulated shared target genes of TLR3 and TLR7 are indicated as grey blocks.

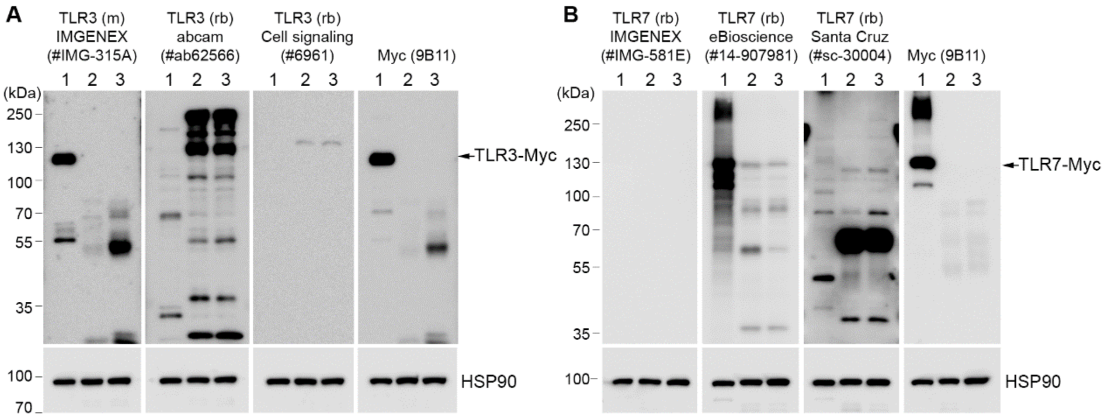

Supplementary Figure 4. Characterization of commercially available TLR3 and TLR7 antibodies.

(**A**) None of the three TLR3 antibodies specifically recognized endogenous TLR3 protein. Lane 1: TLR3-Myc overexpressing HEK293T cell lysate, as a positive control. Lane 2: WT spleen lysate. Lane 3: *Tlr3*$^{-/-}$ spleen lysate. (**B**) Only the TLR7 antibody purchased from eBioscience presented an extract signal (between 55-70 kDa) from WT spleen lysate, presumably representing the cleaved N-terminal fragment of TLR7. Lane 1: TLR7-Myc overexpressing HEK293T cell lysate, as a positive control. Lane 2: WT spleen lysate. Lane 3: *Tlr7*$^{-/y}$ spleen lysate. HSP90 was used as a loading control.

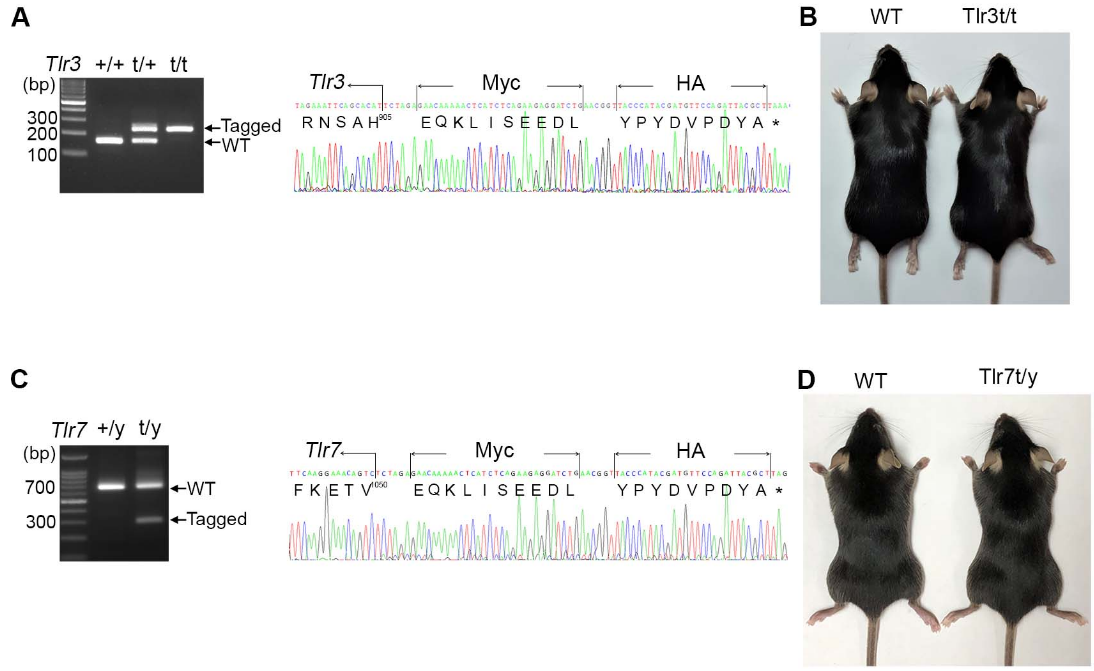

Supplementary Figure 5. Dual tagging of Myc and HA cassettes at the C-terminal ends of the *Tlr3* and *Tlr7* genes in mice.

(**A**) Genotyping results for WT (+/+), *Tlr3*$^{t/+}$, and *Tlr3*$^{t/t}$ mice. Primers Tlr3-Fw and Tlr3-Rv were used for genotyping the tagged *Tlr3* mice. Right: Sequencing result for the Myc-HA-tagged *Tlr3* gene. (**B**) There is no obvious difference in global appearance of WT and *Tlr3*$^{t/t}$ mice. (**C**) Genotyping results for WT (+/y) and *Tlr7*$^{t/y}$ mice. Primers (Tlr7-Fw, Tlr7-Rv and HA-Rv) were used for genotyping the tagged *Tlr7* mice. Right: Sequencing result for the Myc-HA-tagged *Tlr7* gene. (**D**) There is no obvious difference in global appearance of WT and *Tlr7*$^{t/y}$ mice.

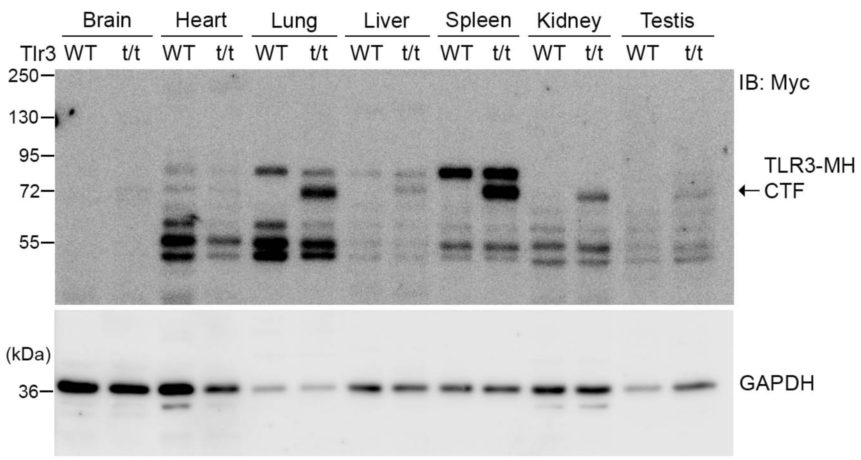

Supplementary Figure 6. Anti-Myc tag antibody does not clearly detect endogenous TLR3-MH proteins in immunoblotting (IB).

The lysates used in Figure 2A were also analyzed using another anti-Myc antibody (9B11). However, in contrast to the results using anti-HA antibody, the Myc tag antibody did not detect full length TLR3-MH, though signal for the C-terminal fragment (CTF) of TLR3-MH were observed for spleen, lung and kidney lysates. Therefore, we used anti-HA antibody to detect dual Myc-HA-tagged TLR3 and TLR7 in our analyses. WT: wild-type, CTF: C-terminal fragment of TLR3.

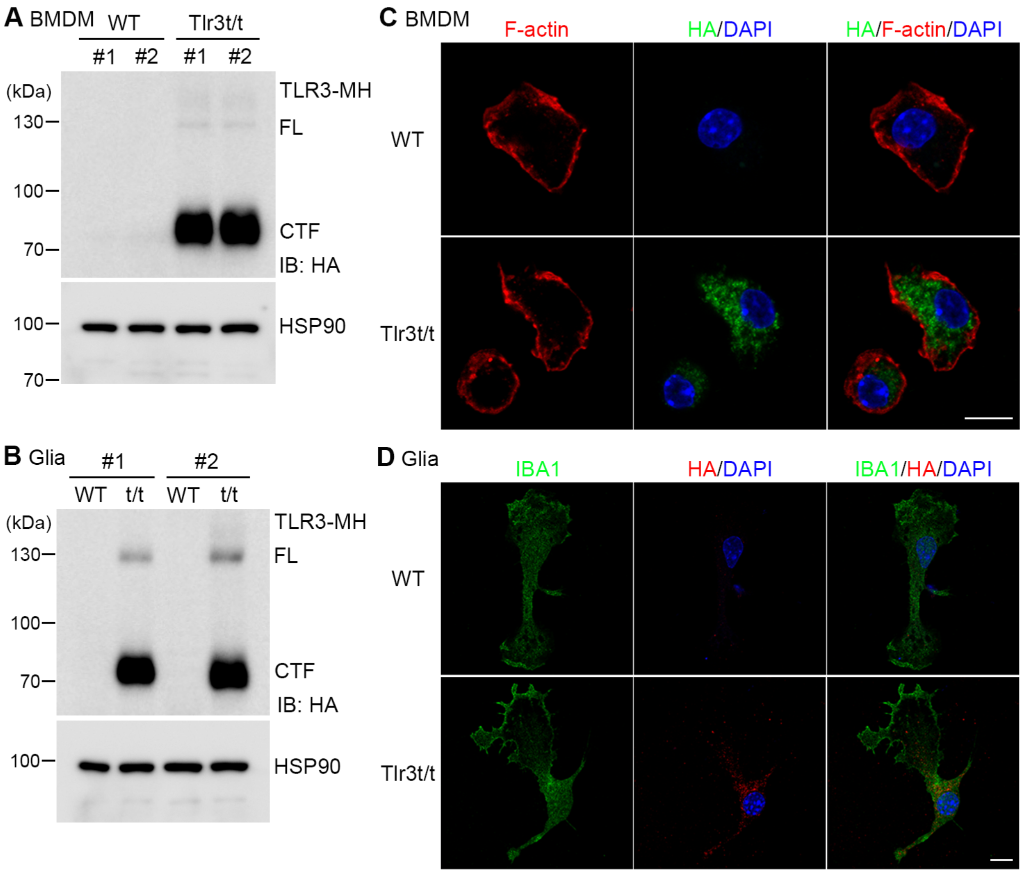

Supplementary Figure 7. The expression pattern and subcellular localization of TLR3-MH protein in primary cell culture.
(**A**, **B**) Detection of TLR3-MH proteins in cultured BMDMs and glial cells of WT and *Tlr3*$^{t/t}$ mice using anti-HA antibody (3F10). HSP90 was used as a loading control. (**C**) Visualization of TLR3-MH protein expression in BMDMs using immunofluorescence staining with anti-HA antibody (C29F4). F-actin revealed by phalloidin staining was used to outline the cell morphology of BMDMs. (**D**) Visualization of TLR3-MH protein expression in microglia using immunofluorescence staining with anti-HA antibody (16B12). IBA1 was used as a microglia marker. Counterstaining with DAPI was performed to label the cell nuclei. Scale bar, 10 μm.

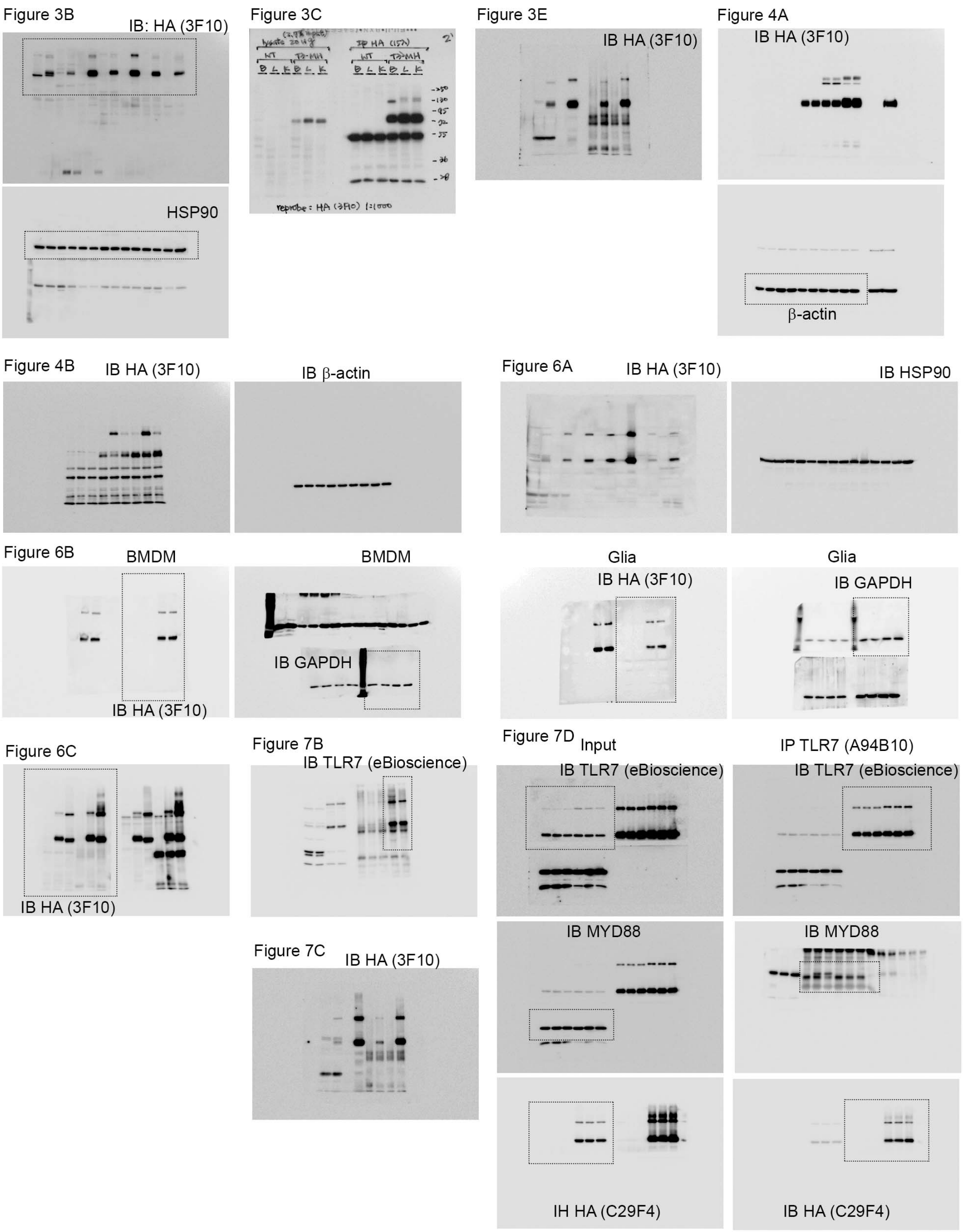

Supplementary Figure 8. Entire images of immunoblots shown in Figures.

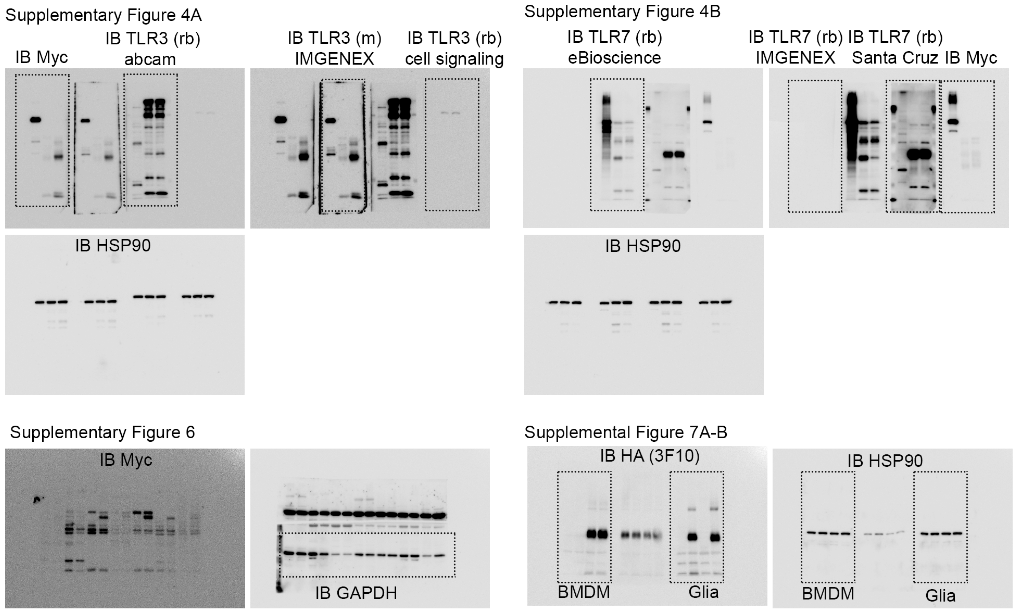

Supplementary Figure 9. Entire images of immunoblots shown in Supplementary Figures 4, 6, 7.

Supplementary Table 2. Antibodies used in the current report

Antibody	Brand and cat.#	Host and clone #	Application
HA	Roche (11867423001)	Rat mAb (3F10)	IB (0.1-0.2 μg/ml)<br>IP (1:250)
HA	Cell Signaling (3724)	Rabbit mAb (C29F4)	IB (0.5-1 μg/ml)<br>IF (1 μg/ml)<br>IP (1:250)
HA	Abcam (ab130275)	Mouse mAb (16B12)	IF (1:500)
Myc	Cell Signaling (2276)	Mouse mAb (9B11)	IB (1:1000)
TLR3	Dr. Kensuke Miyake	Mouse mAb (PaT3)	IP (1:250)
TLR3	IMGENEX (IMG-315A)	Mouse mAb (40C1285.6)	IB (1:500)
TLR3	Cell Signaling (6961)	Rabbit mAb (D10F10)	IB (1:500)
TLR3	Abcam (ab62566)	Rabbit pAb	IB (1:500)
TLR7	Dr. Kensuke Miyake	Mouse mAb (A94B10)	IP (1:250)
TLR7	IMGENEX (IMG-581E)	Rabbit pAb	IB (1:200)
TLR7	eBioscience (14-9079-81)	Rabbit pAb	IB (1:1000)
TLR7	Santa Cruz (sc-30004)	Rabbit pAb	IB (1:200)
IBA1	Wako (019-19741)	Rabbit pAb	IF (1:500)
GFAP	Millipore (MAB3402)	Mouse mAb (GA-5)	IF (1:500)
MYD88	R&D Systems (AF3109)	Goat pAb	IB (0.5 μg/ml)
β-actin	Sigma (A5316)	Mouse mAb (AC74)	IB (1:5000)
HSP90	Dr. Chung Wang	Rabbit pAb	IB (1:4000)
GAPDH	Santa Cruz (sc-25778)	Rabbit pAb	IB (1:1000)
Phalloidin-Alexa546	Invitrogen (A22283)		IF (1:500)

* mAb: monoclonal antibody; pAb: polyclonal antibody; IB: immunoblotting; IF: immunofluorescent staining; IP: immunoprecipitation.

Supplementary Table 3. Software used in the current report

Software and algorithms	Company or website
Prism 8	GraphPad
Hisat2	http://daehwankimlab.github.io/hisat2/
featureCounts	in Bioconductor Rsubread package
edgeR	http://bioconductor.org/packages/release/bioc/html/edgeR.html
R	https://www.r-project.org/
CLC	http://www.clcbio.com
Metascape	http://metascape.org/gp/index.html#/main/step1
ImageJ	https://imagej.nih.gov/ij/

Supplementary Table 4. Primer pairs and UPL probes for quantitative RT-PCR

Gene	Primer pairs	UPL Probe #
Tlr3	F: GATACAGGGATTGCACCCATA<br>R: TCCCCCAAAGGAGTACATTAGA	26
Tlr7	F: TGATCCTGGCCTATCTCTGAC<br>R: CGTGTCCACATCGAAAACAC	25
Il-6	F: GCTACCAAACTGGATATAATCAGGA<br>R: CCAGGTAGCTATGGTACTCCAGAA	6
Il-1β	F: AGTTGACGGACCCCAAAAG<br>R: AGCTGGATGCTCTCATCAGG	38
Tnfα	F: TTGTCTTAATAACGCTGATTTGGT<br>R: GGGAGCAGAGGTTCAGTGAT	64
Ifnβ	F: CACAGCCCTCTCCATCAACTA<br>R: CATTTCCGAATGTTCGTCCT	78
Ccl3	F: TGCCCTTGCTGTTCTTCTCT<br>R: GTGGAATCTTCCGGCTGTAG	40
Tlr2	F: GGGGCTTCACTTCTCTGCTT<br>R: AGCATCCTCTGAGATTTGACG	50
Tlr8	F: CAA ACG TTT TAC CTT CCT TTG TCT<br>R: ATG GAA GAT GGC ACT GGT TC	56
Tlr9	F: GAATCCTCCATCTCCCAACAT<br>R: CCAGAGTCTCAGCCAGCACT	79
Irf7	F: TGTGTCCCCAGGATCATTTC<br>R: CCGGCATCACTAGAAAGCAG	107
Myd88	F: TGGCCTTGTTAGACCGTGA<br>R: AAGTATTTCTGGCAGTCCTCCTC	17
Trif	F: CAGCTCAAGACCCCTACAGC<br>R: CTCCCACACAGCCTCGTC	67
Stat3	F: TGCAGCAGCTGGACACAC<br>R: ACGTGGCATGTGACTCTTTG	1
Ccr3	F: GAGCATCAACAACACGTTCC<br>R: TGAAAGTGTGATCTTGGGACA	77
Mettl3	F: GAAACAGCTGGACTCGCTTC<br>R: GCTTCTGGGTTCCTTAAATCC	38
Hdac1	F: AGCTTCACATCAGCCCTTCT<br>R: GGCAGCATCCTCAAGTTCTC	80

Hdac4	F: GCAAGATCCTCATTGTAGACTGG<br>R: GAACATTGGGGTCATTGTAGAAG	3
Casp3	F: GAGGCTGACTTCCTGTATGCTT<br>R: AACCACGACCCGTCCTTT	80
Atg7	F: GCTTTCACCAAAACAGATCCA<br>R: TCGGCTCGACACAGATCAT	1
Hprt	F: CTTCCTCCTCAGACCGCTTT<br>R: GGTTCATCATCGCTAATCACG	95
